# A cell cycle kinase with tandem sensory PAS domains integrates cell fate cues

**DOI:** 10.1038/ncomms11454

**Published:** 2016-04-27

**Authors:** Thomas H. Mann, W. Seth Childers, Jimmy A. Blair, Michael R. Eckart, Lucy Shapiro

**Affiliations:** 1 Department of Developmental Biology, Stanford University School of Medicine, Stanford, California 94305, USA; 2 Department of Biochemistry, Stanford University School of Medicine, Stanford, California 94305, USA; 3 Department of Chemistry, Williams College, Williamstown, Massachusetts 01267, USA; 4 Stanford Protein and Nucleic Acid Facility, Beckman Center, Stanford University School of Medicine, Stanford, California 94305, USA

## Abstract

All cells must integrate sensory information to coordinate developmental events in space and time. The bacterium *Caulobacter crescentus* uses two-component phospho-signalling to regulate spatially distinct cell cycle events through the master regulator CtrA. Here, we report that CckA, the histidine kinase upstream of CtrA, employs a tandem-PAS domain sensor to integrate two distinct spatiotemporal signals. Using CckA reconstituted on liposomes, we show that one PAS domain modulates kinase activity in a CckA density-dependent manner, mimicking the stimulation of CckA kinase activity that occurs on its transition from diffuse to densely packed at the cell poles. The second PAS domain interacts with the asymmetrically partitioned second messenger cyclic-di-GMP, inhibiting kinase activity while stimulating phosphatase activity, consistent with the selective inactivation of CtrA in the incipient stalked cell compartment. The integration of these spatially and temporally regulated signalling events within a single signalling receptor enables robust orchestration of cell-type-specific gene regulation.

## Abstract (web summary)

The membrane-bound kinase CckA controls the activity of the *Caulobacter crescentus* master regulator CtrA, which in turn coordinates asymmetric cell division. Here, the authors show that CckA contains two sensory domains that have distinct sensitivities to fluctuations in cyclic-di-GMP concentration and subcellular niche.

## Graphical abstract

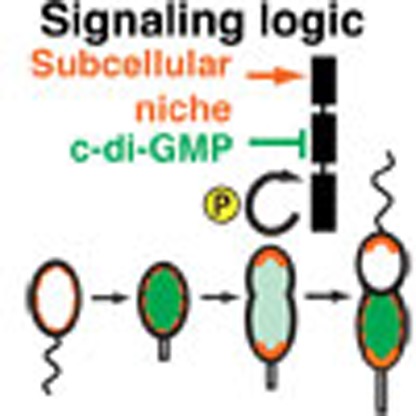

The orchestration of cell-cycle-dependent transcriptional regulation provides the fundamental basis for cellular differentiation and asymmetry throughout all kingdoms of life. A prominent model for asymmetric cell division is the bacterium *Caulobacter crescentus*, which undergoes an asymmetric division during each cell cycle to produce morphologically distinct progeny: a motile swarmer cell and a non-motile stalked cell^1^ (Fig. 1a). Much of the bacterium's cell cycle and developmental coordination involves the periodic activation and inactivation of a master transcriptional regulator, CtrA, which directly controls the expression of at least 90 cell-cycle regulated genes^2^,^3^ and inhibits the initiation of DNA replication^4^ (Fig. 1a). CtrA is active in its phosphorylated state (CtrA∼P), and the phosphorylated form of the regulator is present throughout most of the cell cycle. However, dephosphorylation and proteolysis of CtrA must occur specifically in the stalked cell and in the stalked cell compartment of late predivisional cells to permit the initiation of DNA replication and to activate expression of developmental genes repressed by CtrA∼P (refs 5, 6, 7, 8; Fig. 1a). Critically, CtrA remains phosphorylated in the incipient swarmer compartment, yielding distinct transcriptional profiles for the two daughter cells^2^,^5^.

An essential aspect of the temporal control of gene expression by CtrA is the ability to integrate information from a pathway of phospho-signalling events^9^,^10^,^11^ and second messengers^12^,^13^,^14^,^15^ that report on cell cycle progression. These signals ultimately converge on the hybrid histidine kinase CckA, which regulates CtrA activity and stability through a multifaceted phosphorelay. CckA contains an N-terminal sensory region (comprising two unique PAS domains, PAS-A and PAS-B), a catalytic core (comprising both a dimerization and histidine phosphorylation domain (DHp) and an ATP-binding catalytic assisting (CA) domain), and a C-terminal receiver domain (RD) that indirectly shuttles phosphate to CtrA through the histidine phosphotransferase ChpT (refs 16, 17; Fig. 1b). Kinase activity increases in concert with the accumulation of CckA at the new pole of predivisional cells^18^,^19^,^20^ (Fig. 1a, orange arcs). A striking feature is that CckA remains specifically inactivated when localized at the stalked cell compartment, even in predivisional cells in which CckA accumulates at both the new and old-cell poles^21^. This finding implies a mechanism for the cell-type-specific deactivation of CckA that is independent of CckA density.

CckA is a bifunctional protein, also serving as a cell-cycle-dependent phosphatase for CtrA∼P via back-transfer through ChpT to the RD (ref. 22). CckA subsequently hydrolyses the resulting phospho-aspartate moiety^22^ (Fig. 1c). This phosphatase activity is critical for inhibition of CtrA, which is in turn required for the initiation of chromosome replication^22^. The fact that CckA exhibits phosphatase activity only at specific stages of the cell cycle suggests that this effect is regulated through an active control mechanism. Furthermore, the dynamic regulation of CckA kinase and phosphatase activity suggests that two or more independent regulatory mechanisms control CckA function^23^. Because of the well-established role that PAS sensory domains play in actively controlling CckA-like histidine kinases, and because this regulation often occurs through interactions with small molecules or other proteins^24^,^25^,^26^, we hypothesized that CckA's PAS domains control switching between these bifunctional states.

Here, we interrogate the role of the N-terminal tandem-PAS sensory domains in intramolecular regulation of the bifunctional CckA kinase activity *in vivo* and *in vitro*. By reconstituting active CckA on proteoliposomes, we show that CckA kinase activity is regulated by its surface density and that c-di-GMP independently inhibits CckA kinase function and stimulates CckA phosphatase activity. One of the two PAS domains modulates CckA surface density activation *in vitro* and its subcellular accumulation *in vivo*, while the other mediates c-di-GMP inhibition of CckA and is required for subcellular accumulation at the new cell pole. We present a model in which a tandem-PAS sensor logically wires spatially and temporally independent regulatory mechanisms to enable cell-cycle-dependent changes in a hybrid histidine kinase.

## Results

### PAS domains regulate CckA localization and activity *in vivo*

Structural homology predictions indicated that CckA contains two N-terminal PAS domains, which frequently regulate histidine kinase activity^24^,^27^. To probe the regulatory effects of the PAS domains on CckA activity, we constructed a set of *Caulobacter* strains that contained CckA-eYFP variants with the two PAS domains individually deleted. The CckA-eYFP variants were expressed under their native promoter on a low-copy replicating plasmid as the sole copy of CckA (Fig. 2a). Western blots confirmed stable expression of the plasmid-borne WT and PAS-mutant CckA-eYFP constructs (Supplementary Fig. 1). Strains in which either PAS-A or PAS-B were deleted from CckA exhibited fitness defects, which we quantitated as an increase in cell lengths in the mutant strains (Fig. 2b). Notably, deletion of either PAS domain resulted in a substantial reduction of swarmer-sized cells (2–3 μm), and increase in cell populations with heterogeneous lengths, including 10% of cells with lengths of 8 μm or longer. This filamentation defect is commonly observed with strains that contain misregulated CtrA, due to CtrA's regulation of a small set of genes that control cell division^2^. We were unable to recover a ΔPAS-A ΔPAS-B mutant as the sole copy of CckA, suggesting that disruption of both PAS domains simultaneously is lethal.

To determine if this fitness defect was due to the inability of the CckA mutants to properly localize to the new cell pole, we assayed the impact of the PAS domain deletions on CckA subcellular localization (Fig. 2c). Notably, CckA expressed from low-copy plasmids has been previously reported to bias CckA subcellular localization towards the stalked cell pole as compared with chromosomally produced CckA (ref. 21). Indeed, we observed that when wild-type CckA-eYFP is expressed from a low-copy plasmid under its native promoter, 47% of cells demonstrated bipolar localization, with 24% localized at the stalked pole and 29% of cells diffusely localized, consistent with previous reports^21^. We used a marker of the stalked pole, StpX-mCherry (ref. 28), to assign monopolar cells to the stalked pole (Supplementary Fig. 2). In contrast, both the CckA ΔPAS-A and the ΔPAS-B strains exhibited aberrant, but distinct localization patterns (Fig. 2a,c). Deletion of PAS-A prevented CckA from accumulating at either cell pole (99% of cells contain diffuse CckA), while deletion of PAS-B specifically inhibited localization to the new cell pole with a reduction from 47% bipolar to 6% bipolar. Similarly, fluorescent images of merodiploid strains revealed that WT CckA and CckA ΔPAS-B localized robustly to the cell poles in predivisional cells, whereas CckA ΔPAS-A-ΔPAS-B and CckA ΔPAS-A remained diffuse (Supplementary Fig. 3). Together, these data suggest distinct roles in regulating CckA: PAS-A is required for accumulation at either cell pole, while PAS-B promotes subcellular recruitment to the new cell pole.

Previously, Angelastro *et al*. observed that deleting residues 80–149 within PAS-A also caused a reduction in CckA polar localization and an increase in patchy localizations throughout the cell body^21^. Here, removal of residues 70–196 within PAS-A resulted in diffuse localization of CckA-eYFP. Homology modelling of PAS-A suggested that residues 152–180 contain a portion of the central β-sheet of PAS-A (ref. 29), which may be critical for CckA subcellular localization (Supplementary Fig. 4). A decrease of CckA ΔPAS-B bipolar localization relative to WT CckA suggests that PAS-B may contribute to proper CckA localization (Fig. 2c, N at least 200 cells each). These results indicate that PAS-A is required for both CckA density-dependent activity *in vitro* and for subcellular accumulation at the cell poles *in vivo.*

Previous studies have linked CckA subcellular localization patterns to CckA-CtrA pathway activation^19^,^21^,^30^. Accordingly, we assayed CckA pathway activity in the PAS-mutant strains by performing quantitative reverse transcription PCR (RT-qPCR) to measure the expression for three essential CtrA-controlled genes, ccrM, sciP and divK, relative to an internal control, Rho, not under CtrA control^31^ (Supplementary Fig. 5). For both ΔPAS-A and ΔPAS-B strains, we observed a 2–8-fold reduction in the expression of each CtrA regulated gene, consistent with a loss of CckA kinase activity (Fig. 2d). Together, these data are consistent with previous work indicating that CckA activity *in vivo* depends on its ability to accumulate at the new cell pole^19^.

### CckA autokinase activity on liposomes is density dependent

**Figure 1:**
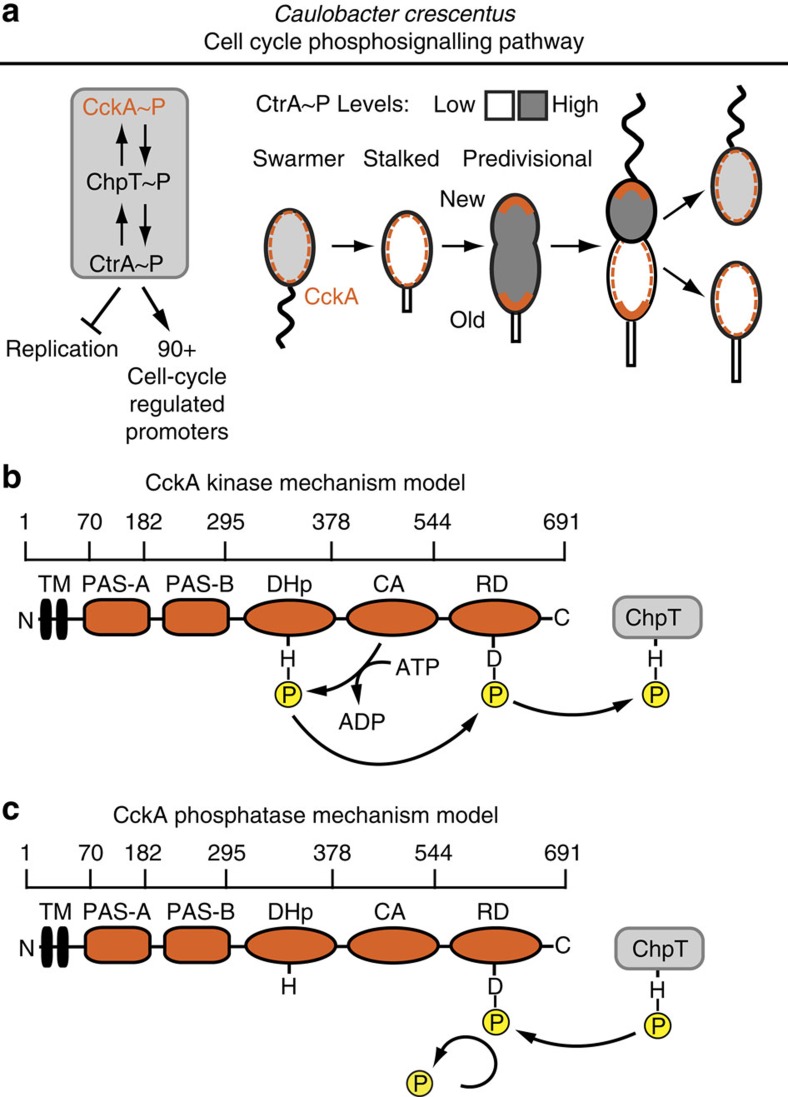
The CckA-ChpT-CtrA signalling pathway directly regulates cell cycle progression in *Caulobacter crescentus*. (**a**) The hybrid histidine kinase (HK) CckA authophosphorylates and subsequently transfers the phosphate through the phosphotransfer protein ChpT to the master regulator CtrA (grey box). Through ChpT, CckA is bifunctional as both a kinase and phosphatase of CtrA. Active CtrA∼P (grey shading) regulates the transcription of more than 90 cell cycle regulated genes and inhibits the initiation of DNA replication. On subcellular localization to the old and new cell poles, the sensor histidine kinase CckA (orange) autophosphorylates and thus activates CtrA∼P. Deactivation of CtrA∼P, enabling DNA replication, occurs in early stalked cells and the stalked cell compartment of predivisional cells via two mechanisms: dephosphorylation and proteolysis. (**b**) A schematic of CckA illustrates its domain architecture and kinase function. The numbered line above CckA indicates residue cutoffs for each of the domains. CckA contains two transmembrane helices at the N-terminus that have been truncated for *in vitro* experiments. Two unique PAS domains, A and B, comprise an N-terminal sensory region. The catalytic core of CckA features a DHp domain, the site of histidine autophosphorylation and an ATP-binding CA domain. The RD shuttles phosphate from the active site histidine through a conserved aspartate to the downstream phosphorelay protein ChpT. (**c**) The schematic of CckA illustrates a model for its phosphatase function. Phosphate can be removed from the conserved aspartate in the RD, allowing CckA to act as a phosphatase for the CtrA pathway. A conserved DxxN residue motif adjacent to the conserved phosphorylatable histidine in the DHp domain assists in catalysis.

It has been shown *in vivo* that CckA phosphorylation levels are high when localized to the poles of predivisional cells, while CckA∼P levels are low when the protein is diffused in daughter cells^15^,^18^. To test the hypothesis that CckA kinase activity is stimulated in a density-dependent manner on the surface of cell membranes, we tethered the cytosolic region of CckA (residues 70–691, henceforth ‘WT', Fig. 1b) to liposomes *in vitro* via an N-terminal His_6_-tag. Liposomes contained 90% di-oleoyl-phosphatidyl glycerol lipids by mass to mimic the lipid composition of *Caulobacter*'s highly negatively charged membrane, which contains mostly phosphatidyl glycerol headgroups^32^. The remaining 10% was composed of nickel-chelated lipids (DGS-NTA) to recruit His-tagged CckA lacking its transmembrane domain^33^.

To investigate the effect of CckA surface density on autophosphorylation activity, we measured the activity at different CckA densities, varying from ∼80 to 800 CckA molecules/liposome (see Supplementary Note 1 for calculation of CckA molecules/liposome from the lipid:CckA mass ratio^34^,^35^,^36^). For the CckA autophosphorylation assay, we fixed the concentration of CckA at 5 μM and added increasing amounts of liposomes, and then incubated the CckA liposomes in the presence of [γ−^32^P] ATP for 3 min. Radiolabelled CckA was then separated from unconsumed [γ−^32^P] ATP by blotting onto nitrocellulose and washing in mildly basic buffer to prevent hydrolysis of the phosphohistidine. CckA activity increased with increasing surface density (Fig. 3a). Densely packed CckA (∼800 molecules/liposome) exhibited ∼10-fold higher autophosphorylation at the assay end point than CckA in the sparsely packed state (∼80 molecules/liposome). This transition could be fit to a sigmoidal curve with a K-half of 340 CckA molecules/liposome. A similar trend existed for the initial rates of kinase activity from 0 to 3 min for a range of CckA densities (Supplementary Fig. 6). These results indicate that the kinase activity rate is enhanced by increases in density of CckA on liposomes ranging from fully activated at high density to fully repressed kinase activity at low density.

### PAS-A promotes autokinase activity on liposomes *in vitro*

**Figure 2:**
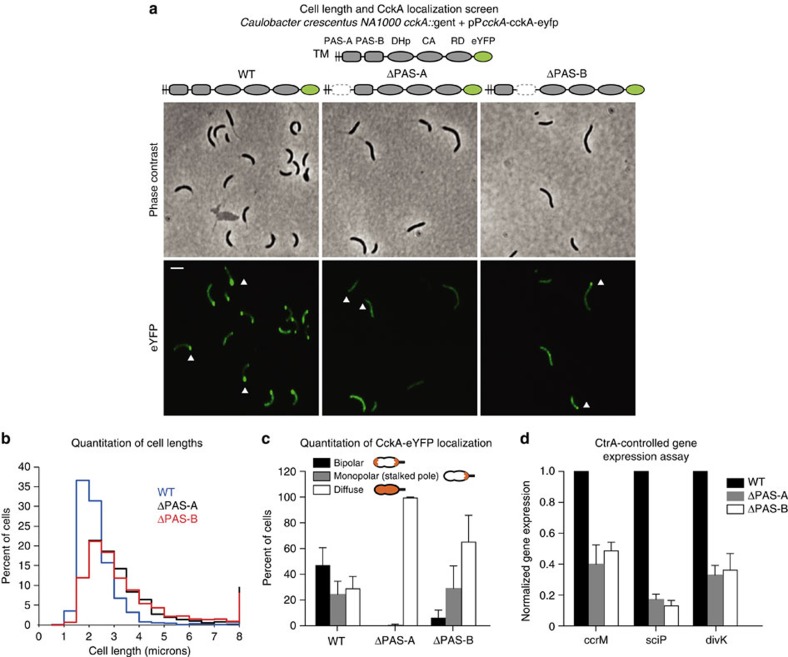
PAS domains regulate CckA localization and activity *in vivo*. (**a**) A cell length and subcellular localization assay shows that deletion of CckA's PAS domains impact both cell length and CckA's subcellular distribution. In each strain, the chromosomal *cckA* locus was replaced with a gentamycin cassette, and the stalked-pole marker protein StpX was expressed with an mCherry tag. CckA-eYFP constructs were expressed from the *cckA* native promoter on a low-copy replicating plasmid. White arrowheads indicate the stalked pole in the eYFP images. Scale bar is the same for each field, 2 μm. (**b**) Deletion of either PAS domain causes an increased cell-length phenotype. Cell lengths were grouped in 0.5 μm bins, with cells >8 μm binned together, and represented as a percentage of the total population. N>1,000 cells for each strain. (**c**) PAS-A is necessary for localization to the cell poles, and PAS-B regulates new pole localization *in vivo*. Quantitation of the proportion of cells in which CckA-eYFP localized to both cell poles (black bar), formed a single focus at the stalked pole (grey bar) or remained diffuse over the membrane (white bar). A stpX-mCherry label was used to definitively assign the stalked pole CckA accumulations. Deletion of PAS-B specifically depleted the new-cell-pole population while maintaining stalk cell pole localization. Deletion of PAS-A totally abrogated subcellular accumulation at both poles. Fewer than 5% of cells in any strain had monopolar accumulations of CckA-eYFP at the new cell pole. Error bars represent the s.d. across individual fields of view, with at least 200 cells counted for each mutant. (**d**) RT-qPCR assays of the expression from CtrA-controlled promoters *ccrM*, *sciP* and *divK* show that deletion of either PAS domain results in decreased gene expression for all three promoters. Expression was measured for a separate amplicon within each of the three genes, and expression of each gene was normalized to an internal standard, an amplicon within Rho. Error bars represent the s.d. of three biological replicates, each composed of at least two technical replicates.

**Figure 3:**
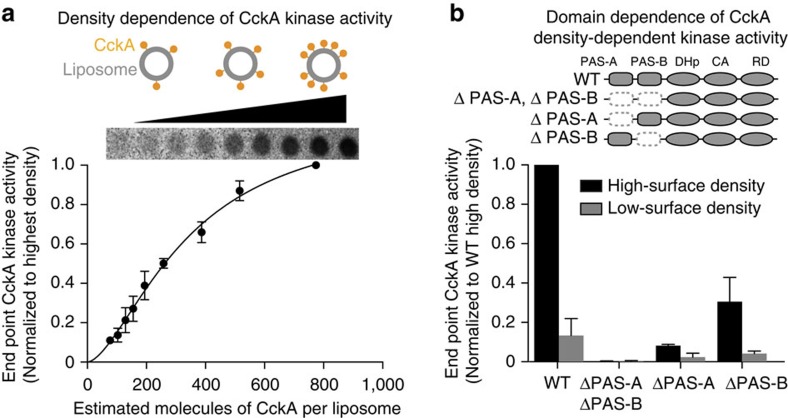
CckA activity on liposomes is density dependent. (**a**) CckA autophosphorylation activity was assayed on liposomes. A fixed amount of 5 μM CckA was pre-incubated with increasing amounts of liposomes to obtain surface densities ranging from ∼80 to 800 CckA molecules per liposome. Autophosphorylation was initiated on addition of [γ−^32^P] ATP and allowed to proceed for 3 min. Samples quenched in SDS sample buffer were assayed using the nitrocellulose membrane capture assay, followed by phosphorimaging. The activity of each CckA surface density state is normalized to the highest density condition. Representative data is shown above the graph. These data were fit to a sigmoidal curve described in the Methods section. (**b**) Assays of the kinase activity levels of CckA PAS domain truncations at maximum surface density (∼1,100 CckA per liposome) and low-surface density (∼100 CckA per liposome) showed that the CckA PAS sensor, particularly PAS-A, is required for CckA activity on liposomes. CckA activity levels were normalized to the WT activity at maximum density. Each CckA construct's domain architecture is shown as a cartoon accompanying its data. Dark grey shading of a domain indicates that the domain is present, while a dashed, white box indicates that the domain is absent. Error bars in **a**,**b** represent the range of two experiments.

To determine if the sensory domain of CckA contributes to the density-dependent kinase activation, we systematically deleted the two PAS domains either individually or in combination and measured kinase activity at high density (∼1,100 CckA/liposome) versus low density (∼100 CckA/liposome; Fig. 3b). Each construct had kinase activity in solution, indicating that constructs were well folded in solution (Supplementary Fig. 7). Removal of the tandem-PAS sensor, ΔPAS-A-ΔPAS-B, completely eliminated kinase activity on liposomes, indicating that the sensor domain is required for kinase activity on liposomes. CckA constructs missing PAS-A exhibited significantly reduced activation of CckA autophosphorylation on liposomes (Fig. 3b). However, CckA ΔPAS-B maintained density-dependent activation, exhibiting kinase activity when densely packed on liposomes and diminished activity when sparsely packed on liposomes. Comparison of ΔPAS-A-ΔPAS-B and ΔPAS-A suggests that PAS-B also plays a minor role in reaching full activation at high density. The subcellular localization and qPCR assays (Fig. 2c,d) indicate that PAS-B plays an additional role in activation of CckA *in vivo* by promoting its localization at the new pole, previously shown to be important for kinase activity^19^. The precise mechanism of density-dependent activation requires extensive further biophysical characterization, but encompasses a variety of mechanisms that include changes in oligomerization state.

### Cyclic-di-GMP inhibits CckA

**Figure 4:**
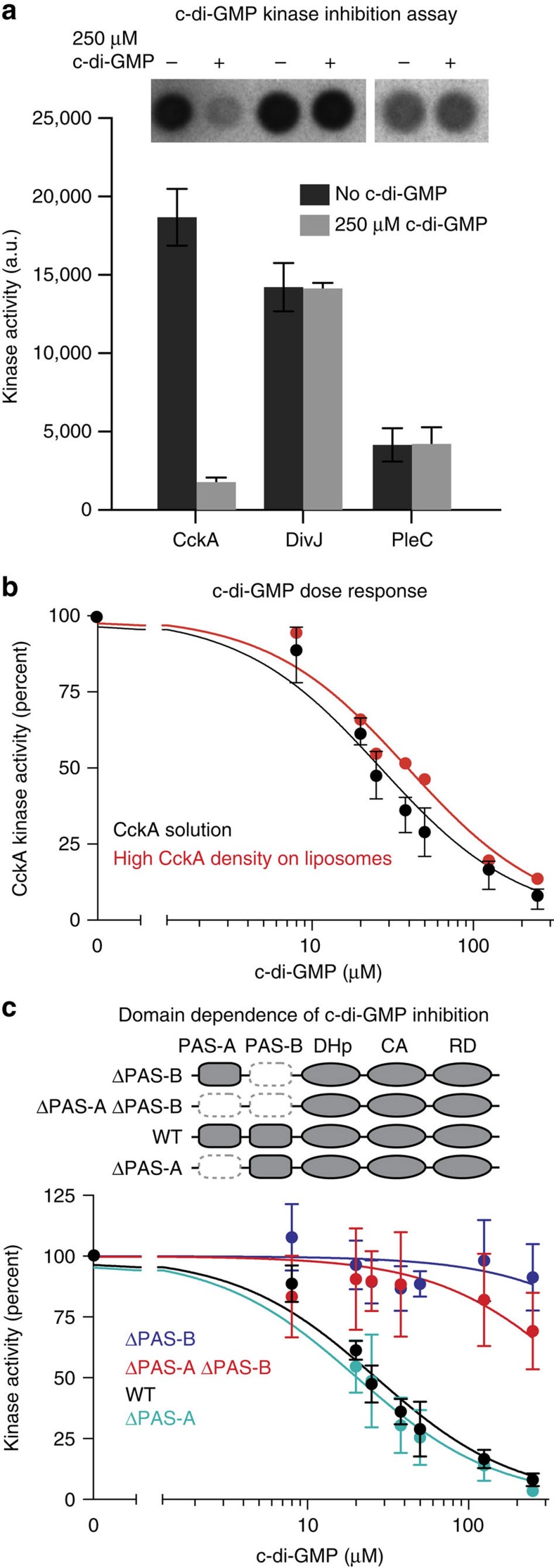
Cyclic-di-GMP specifically inhibits CckA. (**a**) C-di-GMP inhibits CckA autophosphorylation, but does not affect the kinase activity of two other polar-localized cell cycle HKs (PleC and DivJ). Purified CckA, DivJ or PleC at 5 μM was incubated with (grey) or without (black) 250 μM c-di-GMP in the presence of 0.5 mM ATP and 4.2 μCi [γ−^32^P] ATP for 15 min. Samples were quenched into SDS sample buffer, blotted onto nitrocellulose and followed by phosphorimaging. Representative nitrocellulose membrane capture assays are shown above the graph. Error bars represent the s.d. of three experiments. (**b**) The CckA kinase activity dose-response curve in the presence of increasing amounts of c-di-GMP (0–250 μM) was measured in solution and on liposomes. Liposomes were loaded to maximum CckA surface density with ∼1,100 CckA molecules per liposome. Activity is normalized to the zero c-di-GMP condition for CckA in solution and on liposomes. For the liposome data, each point represents the mean of two experiments, and for the solution each point represents the mean of two to five experiments. Error bars correspond to the range of the data and they are smaller than the symbols for several data points. The data were fit to a sigmoidal curve described in the Methods section. (**c**) CckA kinase activity dose-response curve in the presence of increasing amounts of cyclic-di-GMP for various CckA domain deletions. CckA constructs missing PAS-B showed little to no response to c-di-GMP. Dose-response curves were collected and analysed as in Fig. 4b. The set of CckA truncations used to analyse the domain dependence of c-di-GMP inhibition are shown. Dark grey shading indicates that a domain is present, while a dashed, white box indicates that the domain is absent. Error bars represent the s.d. of at least three experiments.

Previous work has shown that CtrA is selectively deactivated on compartmentalization in the stalked compartment of predivisional cells, despite the fact that CckA accumulates at high density at the stalked pole that could promote kinase functions^5^,^21^,^30^. Because the second messenger c-di-GMP has been shown to be asymmetrically distributed to the stalked cell compartment in the predivisional cell, where CckA is inactive, we considered the possibility that c-di-GMP inhibits the kinase function of CckA (ref. 37). To test this possibility, we assayed CckA autophosphorylation *in vitro* using the nitrocellulose membrane capture assay, comparing the amount of CckA phosphorylation after 15 min between reactions with or without 250 μM c-di-GMP (Fig. 4a). C-di-GMP inhibited CckA kinase activity, but did not inhibit two other polar-localized, PAS-containing histidine kinases, DivJ and PleC. Notably, DivJ and PleC lie upstream of CckA in its signalling pathway and also indirectly regulate the available cellular pool of c-di-GMP (ref. 37), suggesting that they serve as feedback targets for c-di-GMP. Thus, c-di-GMP inhibits CckA kinase activity but not that of related polar-localized signalling proteins.

To probe the chemical specificity of the interaction between CckA and c-di-GMP, we assayed CckA autophosphorylation in the presence of a set of chemically similar analogues and signalling molecules (Supplementary Fig. 8). Amongst this set of molecules, the alarmone signal ppGpp is particularly notable because it is a known CtrA regulator^12^,^13^,^14^. C-di-GMP was the only ligand in the set that inhibited CckA *in vitro*, indicating a specific interaction between CckA and c-di-GMP. Additionally, we note that increasing concentrations of c-di-GMP inhibited CckA kinase function in an initial rate assay (Supplementary Fig. 9). Consistent with other c-di-GMP binding proteins^38^, we observed that inhibition of CckA was Mg^2+^-dependent (Supplementary Fig. 10).

By titrating increasing amounts of c-di-GMP into CckA autophosphorylation reactions, we determined that c-di-GMP inhibited CckA with an IC_50_ of 27 μM (95% confidence interval=23–30 μM) in extended kinase assays (Fig. 4b). While this IC-50 is higher than the cell-cycle-dependent intracellular c-di-GMP concentration of 0.1–1.1 μM (ref. 39), recent work demonstrated similar potency for c-di-GMP inhibiting CckA *in vitro*, and physiological relevance for the interaction *in vivo*, suggesting that measurement of this interaction may be further optimized *in vitro*^15^.

We also tested the ability of c-di-GMP to inhibit CckA autophosphorylation at high density on liposomes. By titrating c-di-GMP into liposomes densely packed with CckA, we found that c-di-GMP inhibited CckA on liposomes with an IC_50_ of 39 μM (95% confidence interval=34–45 μM), similar to the IC_50_ observed for CckA in solution (Fig. 4b). This finding implies a signalling logic in which inhibition of CckA by c-di-GMP overrides density-dependent stimulation of autokinase activity. Critically, the ability of c-di-GMP to inhibit CckA in a high-density state *in vitro* is consistent with the observed inhibition of accumulated CckA at the stalked pole *in vivo*^5^,^15^,^21^,^22^.

### Cyclic-di-GMP inhibition of CckA requires the PAS-B domain

To determine the domain dependence of c-di-GMP inhibition of CckA kinase activity, we purified a set of domain-truncated CckA mutants and assayed each for c-di-GMP inhibition (Fig. 4c). Kinase assays using CckA variants missing one or both PAS domains were performed in the presence of increasing concentrations of c-di-GMP. Both WT CckA and CckA ΔPAS-A were robustly inhibited by c-di-GMP, exhibiting IC_50_s of 27 μM (95% confidence interval=23–30 μM) and 22 μM (95% confidence interval=16–26 μM), respectively. However, both constructs missing PAS domain B (ΔPAS-B and ΔPAS-A-ΔPAS-B) were unresponsive to c-di-GMP, suggesting that PAS domain B is necessary in order for c-di-GMP to inhibit CckA autophosphorylation.

### CckA directly binds c-di-GMP through multiple domains

**Figure 5:**
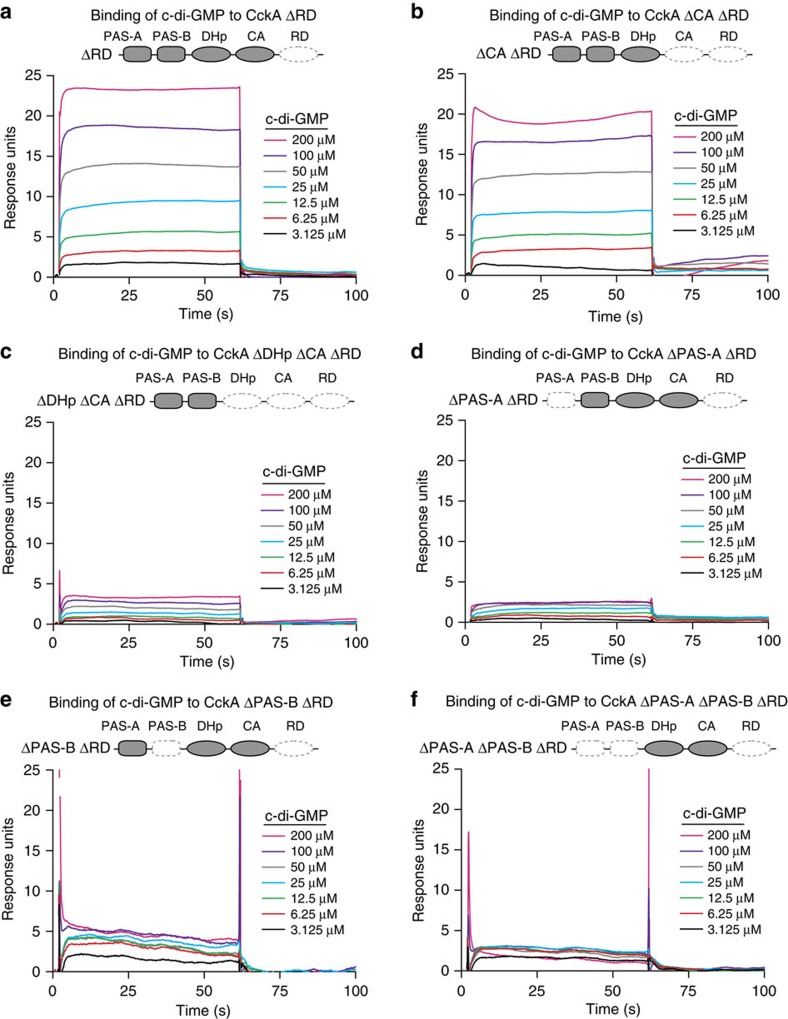
C-di-GMP directly binds CckA in a PAS domain-dependent manner. (**a**) SPR experiments show that CckA directly binds c-di-GMP. Purified CckA ΔRD was tethered to the SPR chip and exposed to increasing concentrations of injected c-di-GMP. The binding period is displayed as response units plotted as function of time (*t*=2–62 s), followed by buffer injection to remove c-di-GMP. Increasing response curves were obtained using the increasing c-di-GMP concentrations shown on the right. (**b**–**f**) Additional SPR experiments examining the interaction of c-di-GMP with (**b**) CckA ΔCA ΔRD, (**c**) ΔDHp ΔCA ΔRD, (**d**) ΔPAS-A ΔRD, (**e**) ΔPAS-B ΔRD and (**f**) ΔPAS-A ΔPAS-B ΔRD.

We performed surface plasmon resonance (SPR) binding experiments to test if c-di-GMP binds directly to a set of CckA variants lacking one or more functional domains. All analysed CckA variants lacked the RD, which causes instability of CckA on the SPR chip. After immobilizing CckA ΔRD on the SPR sensor chip, increasing concentrations of c-di-GMP were injected over the immobilized CckA variants. The concentration-dependent response we observed demonstrates that c-di-GMP directly binds CckA (Fig. 5a). While we cannot rule out the possibility that the RD domain further enhances binding to c-di-GMP, these results show that CckA is capable of binding c-di-GMP without the RD.

SPR experiments also revealed that removal of the ATP-binding CA domain resulted in a minor reduction in c-di-GMP binding capability (Fig. 5b). When we additionally removed the DHp domain (Fig. 5c) or the PAS-A domain (Fig. 5d), we observed significant reductions in binding. Each construct still displayed concentration dependence in response to c-di-GMP and a stable response over the injection period, suggesting that both DHp and PAS-A contribute to c-di-GMP binding. Although the isolated PAS-B domain was unable to bind c-di-GMP, removal of PAS-B from more complete constructs abrogated concentration dependence at c-di-GMP concentrations above 6.25 μM. Additionally, the response curve was unstable over time, indicative of inconsistent or transient interactions between c-di-GMP and CckA lacking PAS-B (Fig. 5e). The instability of the interaction between CckA and c-di-GMP on the removal of PAS-B suggests that PAS-B is necessary for formation of a stable, productive interaction between CckA and c-di-GMP. Simultaneous removal of PAS-A and PAS-B completely eliminated concentration dependence, further suggesting that PAS-A contributes to the stable binding of c-di-GMP (Fig. 5f).

Cumulatively, our results show that c-di-GMP directly binds CckA and inhibits its kinase activity through a complex, multi-domain interaction centred on PAS-B. The PAS-A and DHp domains play accessory roles in c-di-GMP binding, with a minor contribution from the CA domain. These secondary contributions may occur either through direct contacts with c-di-GMP or indirectly by promoting proper dimerization of the PAS-B receptor domain. This dimerization model is consistent with general c-di-GMP receptor function, in which c-di-GMP frequently binds to dimerized GGDEF domains^38^. Critically, though, stable binding of c-di-GMP to CckA and inhibition of CckA autophosphorylation both require PAS-B, implicating this domain as the primary receptor for c-di-GMP on CckA.

### C-di-GMP increases CckA phosphatase activity *in vitro*

Because we found that both surface density and c-di-GMP levels affect kinase activity of CckA, we asked if these two signal inputs could also regulate CckA phosphatase activity. To determine the effect of CckA surface density on phosphatase activity, we allowed CckA to autophosphorylate in solution using radiolabelled [γ−^32^P] ATP, and then added hexokinase and glucose to convert the unconsumed ATP into ADP. CckA∼P was then titrated into solutions containing different amounts of liposomes and the decay of the CckA∼P signal was monitored. We observed that the CckA∼P half-life was ∼25 min over a broad range of CckA surface densities (50–950 molecules/liposome), with a potential increase, albeit mild, in the half-life of CckA∼P at the lowest densities (Supplementary Fig. 11). Cumulatively, these results argue that variation of CckA surface density modulates CckA kinase activity but minimally impacts phosphatase activity.

**Figure 6:**
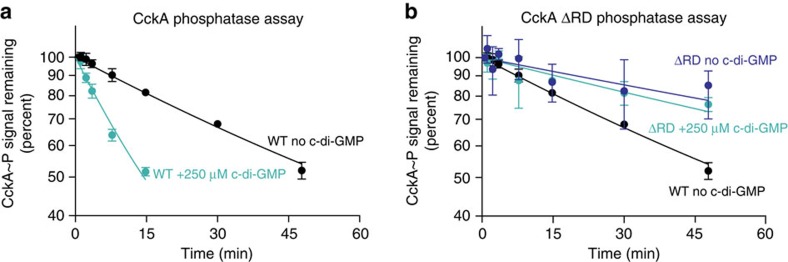
C-di-GMP inhibits CckA by increasing its phosphatase rate. (**a**) Purified CckA∼P was incubated over time (0.5, 1, 2.5, 3.5, 8, 15, 30 and 48 min, rounded to the nearest 0.5 min) with or without 250 μM c-di-GMP. At each time point, samples were quenched into SDS sample buffer, blotted onto nitrocellulose and followed by phosphorimaging. The remaining signal is plotted as a fraction of the initial time point. The data are fit to a single exponential decay with a plateau at 20% signal due to a long-lived subpopulation of CckA∼P. (**b**) C-di-GMP-mediated CckA phosphatase activity depends on presence of the RD. Time course of CckA dephosphorylation is shown in the presence and absence of cyclic-di-GMP. Samples were processed as described in **a**. Error bars in **a**,**b** represent the s.d. of three experiments.

We next tested the effect of c-di-GMP on CckA phosphatase activity by generating CckA∼P *in vitro* and purifying the protein away from ATP, allowing us to monitor the CckA dephosphorylation rate. Incubating CckA∼P with c-di-GMP led to rapid dephosphorylation of CckA∼P, suggesting that c-di-GMP increased the rate of CckA phosphatase activity (Fig. 6a). Because hybrid histidine kinases such as CckA can catalyse the removal of a phosphate from their RDs (refs 22, 40), we examined the effect of incubating CckA∼P ΔRD with c-di-GMP. Critically, we observed that CckA∼P ΔRD dephosphorylation was unresponsive to the addition of c-di-GMP (Fig. 6b). Thus, despite a robust c-di-GMP binding capability, CckA ΔRD is not able to catalyse phosphate hydrolysis from the phosphorylated histidine, consistent with the current understanding of hybrid histidine kinase phosphatase activity^40^ (Fig. 1c). Taken together, these results indicate that CckA phosphatase activity is only affected by the c-di-GMP signal sensed by the proximal PAS-B domain.

## Discussion

**Figure 7:**
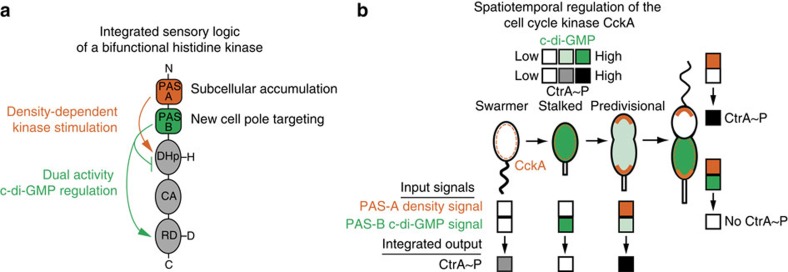
CckA drives cell fate by integrating sensory information in time and space. (**a**) Sensory domains regulate the bifunctional activity of CckA. PAS-A is required for density-dependent regulation of kinase activity on liposomes. PAS-B independently inhibits kinase function while stimulating phosphatase activity at the RD upon binding c-di-GMP. Integrating these two independent regulatory mechanisms enables a spectrum of functional states. (**b**) A schematic of the *Caulobacter* cell cycle showing the subcellular CckA surface density (orange) and the asymmetric distribution of c-di-GMP (green)^37^. Below the cells, coloration of a pair of boxes indicates the strength of the input signals for CckA PAS-A and PAS-B, CckA clustering at the cell poles, and the presence or absence of c-di-GMP during cell cycle progression. Processing the effects on its kinase and phosphatase activity, CckA integrates these signals to a single net activity, reflected as the downstream integrated output, shown in the CtrA∼P output box (greyscale).

Here, we demonstrate that the bifunctional hybrid HK CckA employs a tandem-PAS sensor to integrate two spatially independent signals that exert opposite effects on HK function (Fig. 7a). Domain deletion analysis revealed that PAS-A primarily mediates density-dependent CckA kinase activity on liposomes *in vitro* (Fig. 3b) and subcellular accumulation of CckA at the cell poles *in vivo* (Fig. 2c), whereas the second PAS domain, PAS-B, is required for targeting to the new cell pole and for c-di-GMP-stimulated CckA phosphatase activity (Figs 2, 3, 4, 5, 6).

Temporal separation of peak accumulation of c-di-GMP from peak CckA surface density allows CckA to apply hard-wired tandem-PAS-dependent logic to toggle between three functional states (kinase, inactive or phosphatase) consistent with observed CtrA regulation of cell-cycle progression (Fig. 7b). In swarmer cells, c-di-GMP levels are low and CckA is largely diffuse, resulting in an inert state in which neither phosphatase nor autokinase activities are stimulated^21^,^41^ (Fig. 7b). These cells exhibit intermediate levels of CckA∼P and CtrA∼P (refs 18, 21; Fig. 1a). At the swarmer-to-stalked transition, c-di-GMP levels increase significantly^37^,^41^ and CckA remains delocalized, resulting in the CckA phosphatase-active state^5^,^21^ (Fig. 7b); this is consistent with the observed clearance of CtrA at this transition, occurring simultaneously with dephosphorylation of CtrA∼P (ref. 7). Predivisional cells contain densely packed CckA at the cell poles and relatively low c-di-GMP levels^21^,^41^, yielding a kinase-active state for CckA that is consistent with previous *in vivo* results^18^,^30^. Before cytokinesis, the predivisional cell compartmentalizes, leading to the selective build-up in the stalked compartment of c-di-GMP (switching CckA to the phosphatase-active state^37^,^42^ and leading to CtrA inactivation/clearance), while lower c-di-GMP levels in the incipient swarmer compartment maintain CckA kinase function and stabilize the active, phosphorylated form of CtrA (Fig. 7b). In this way, the unique sensory logic of the tandem-PAS sensor (Fig. 7a) integrates cell cycle fluctuations in CckA density and intracellular cyclic-di-GMP levels to allow for distinct CckA functional states that regulate the activity of the CtrA master transcription regulator during cell cycle progression.

The tandem-PAS sensor further directs cell cycle progression through its domain-specific regulation of CckA localization. CckA lacking PAS-A does not accumulate at either cell pole, while CckA lacking PAS-B has decreased localization to the new cell pole (Fig. 2c). Accumulation of CckA specifically at the new pole is strongly linked to its activity as a kinase^19^, and indeed we observed in gene expression assays that CtrA-controlled genes have reduced expression in strains harbouring these PAS mutants as the sole copy of CckA (Fig. 2d). The loss of ΔPAS-A CckA kinase activity *in vivo* is consistent with its inability to achieve a high-density state at the cell poles. In addition, we also observed that CckA ΔPAS-B led to a reduction in CckA-CtrA pathway activity, despite the fact that this variant was unresponsive to high c-di-GMP concentrations. These results point towards two possible models for the role of PAS-B *in vivo*. First, new pole-specific factors such as the pseudokinase DivL may directly contribute to activating CckA, and the inability of ΔPAS-B to localize to the new pole either directly or indirectly through DivL prevents this activation^19^. Second, extensive genetic studies have indicated that the phosphorylated form of the response regulator DivK, in a complex with the pseudokinase DivL, inhibits CckA (refs 9, 10, 20). This DivK∼P:DivL-dependent inhibition of CckA may be independent of CckA's PAS-B and thus still able to inhibit CckA ΔPAS-B *in vivo*^9^,^15^,^20^.

Previous *in vivo* studies have led several labs to propose that unique subcellular niches (new cell pole, stalked cell pole, diffuse cell body) harbour different CckA functions^19^,^20^,^21^,^22^. Here, we have recapitulated density-specific activation *in vitro* such that CckA kinase is inactive at low density and active at high-surface density (Fig. 3a). Thus, a density-dependent sensor allows a mechanism of varying CckA activity within subcellular regions, thereby promoting kinase function only when CckA is densely packed at the cell pole and preventing activity while diffuse throughout the cell. CckA can accumulate at both poles, more frequently at the new cell pole^21^. It remains unclear what mechanism underlies density-dependent activation of CckA, which encompasses a variety of models that includes modulation of CckA's oligomerization state.

In addition, the mechanisms for difference in function between CckA at the two cell poles before compartmentalization of the predivisional cell remain unclear, because the fast-diffusing c-di-GMP small molecule would be expected to impact both clusters equally^21^,^22^,^43^. However, once compartmentalization occurs, high levels of c-di-GMP in the stalked cell compartment can inhibit CckA kinase activity while low levels in the swarmer compartment permit kinase activity of dense CckA at the new pole. More broadly, density-dependent modulation of signalling molecule activity appears to be a widespread phenomenon that is observed in T-cells, chemotaxis arrays and circadian clock proteins alike^44^,^45^,^46^.

Here, we observe that the PAS-A sensory domain is important not only for promoting density-dependent CckA activity *in vitro*, but is also necessary for accumulation of CckA at the cell poles *in vivo*. Structural studies have demonstrated that PAS domains can form protein–protein contacts using several distinct interfaces within the PAS domain^47^. These interactions could contribute to the PAS-A-dependent activity at high density by promoting higher-order oligomerization of nascent CckA dimers. Additionally, PAS-A may form contacts with factors known to be involved in polar localization of CckA, such as the polar pseudokinase DivL, the polar phosphatase PleC, the localization factor PodJ or the PopZ polar matrix protein^19^,^48^,^49^. The density-dependent increase in CckA activity reported here provides an explanation for the *in vivo* connection between subcellular accumulation and activity, and implies a control mechanism for coordinating sequential cell cycle events through the assembly of a signalling complex at the new cell pole.

Through an elegant set of genetic experiments, Lori *et al*.^15^ demonstrated that c-di-GMP plays a direct role in inhibiting CckA activity *in vivo* and provided *in vitro* biochemical evidence suggesting that c-di-GMP interacts with CckA via the CA domain. Here, through a comprehensive set of SPR binding experiments, we have demonstrated that CckA binding of c-di-GMP employs PAS-B as a primary receptor with additional binding contributions from PAS-A and DHp along with minor contributions from the CA domain (Fig. 5). Further, PAS-B is necessary for inhibition of CckA by c-di-GMP, supporting the notion that PAS-B is the primary receptor for c-di-GMP (Fig. 4c). Previous cross-linking biochemical studies from Lori *et al*.^15^ combined with our SPR binding studies collectively suggest that c-di-GMP may lie at an interface between PAS-B and the CA domain. Future high-resolution structural studies will help reveal the precise c-di-GMP-CckA binding mechanism.

Understanding how c-di-GMP triggers the switch from kinase to phosphatase states will also broadly illuminate signal transduction mechanisms in hybrid HKs. Here, we showed that c-di-GMP inhibition of CckA kinase activity and stimulation of phosphatase activity not only requires PAS-B, but also requires the RD (Fig. 6). Critically, the RD is not required for c-di-GMP binding, but instead is required for c-di-GMP-mediated phosphatase activity. Our domain deletion analysis implicates the conserved aspartate of the RD as the regulated substrate for desphosphorylation^22^. This phosphatase mechanism is consistent with previous *in vitro* work which showed that mutations in the DHp domain can impair CckA phosphatase activity directed against the RD (ref. 22). Indeed, the DHp domain contains a conserved DxxN motif that has previously been implicated in regulating HK phosphatase activity towards RR proteins^40^. Accordingly, we suggest that this motif is likely also functional within the context of chimeric hybrid histidine kinases.

Two discrete domains separate PAS-B and RD, raising the question of how this intramolecular regulation occurs despite the physical separation of these domains. One hypothesis is that c-di-GMP binding in PAS-B alters the DHp four-helix bundle configuration relative to RD to promote interaction between the catalytic residues and the phospho-aspartate substrate. This putative mechanism is analogous to models of allosteric kinase regulation in which a signal modulates kinase activity by inducing a conformational change in the DHp domain relative to the CA domain^24^,^50^,^51^,^52^,^53^. Alternatively, PAS-B may directly propagate its signal to the RD through lateral contacts to modulate the RD-DHp interaction in a way that stimulates phosphatase activity. Given the widespread distribution of PAS domains and c-di-GMP as signalling modules in bacteria^24^,^54^, this work hints that this ligand–receptor pair may be prevalent in many other species. Future studies will illuminate whether this interaction between PAS and c-di-GMP is widely conserved and will shed light on the molecular mechanism that stimulates phosphatase activity in a hybrid HK.

Multi-sensor HKs are critical signalling components of many complex developmental programs in bacteria including plant pathogenesis, sporulation, and biofilm formation^53^,^55^,^56^. Nearly one third of PAS-containing proteins contain two or more PAS domains^25^, but the signal transduction of multi-sensor HKs remains poorly understood. While multiple single-sensor HKs can operate on the same target protein^57^, leading to integration of signals at the RR level, the presence of multiple sensory domains within a single kinase allows for the direct integration of multiple signals at the point of HK phosphorylation. An advantage of integrating signals at the point of receptor activation is that it ensures receptor stoichiometry and eliminates noise from variation in signal-receptor copy number, while allowing complex decisions to be made based on multiple spatially regulated signal inputs^58^.

Several studies have demonstrated the interchangeability of single-sensory domains in bacterial histidine kinases^19^,^59^. However, the complex interplay between sensory domains is evident in the *Agrobacterium tumefaciens* multi-sensor kinase VirA, whose two input signals drive output akin to an AND logic gate^60^. Studies of the *Fremyella diplosiphon* IflA cyanobacteriochrome photorecptor indicate that domain interactions between adjacent sensory domains increases the color-sensing capabilities relative to isolated photosensory domains^61^. These results indicate that when sensors are placed in tandem, they can function in an interdependent manner that enhances information processing capabilities, as opposed to sensing functions being completely separable and modular. Here, we observed that each sensory domain in CckA serves a primary role of driving density-dependent regulation or c-di-GMP sensing (Fig. 7), but the adjacent domains to each sensor appear to mildly influence each other's function (Figs 3,5). Moreover, the PAS-B based sensing of c-di-GMP was dominant over the PAS-A based surface density signal, reflective of synthetic multi-sensor kinases in which the sensory domains in closer proximity to the effector domain have a larger impact on kinase catalytic output^59^.

Our analysis of CckA expands the repertoire of signalling schemes of multi-PAS kinases by demonstrating how different PAS domains can toggle distinct catalytic functions to achieve multiple functional states (kinase, phosphatase and inactive). Collectively, these studies of multi-sensor kinases suggest that partial integration of sensory domain functions is crucial for both optimization and variation of information processing logic. The combination of sensor domains throughout evolution has likely created diverse and as-yet-undiscovered signal integration schemes that enable complex bacterial decision-making.

## Methods

### Cloning

CckA domain cutoffs were assigned using the HHpred protein homology web server^27^. CckA constructs containing PAS-A but missing PAS-B (ΔPAS B and ΔPAS B-ΔRD) were generated by fusing the signal transmission region C-terminal of PAS-B (residue 296 onward) to a conserved DAS sequence motif (residues 180–182) that marks the end of a PAS domain in many kinases^24^.

Plasmids for this study were created through restriction ligation cloning or Gibson assembly designed through the J5 cloning system^62^. Plasmids were transformed into *E. coli* via heat shock and into *C. crescentus* (strain NA1000) via electroporation. An example for how to read the tables to understand plasmid construction is given for restriction ligation and Gibson cloning.

The plasmid pJAB41 was generated via restriction ligation cloning and permits overexpression of CckA ΔDHp-ΔCA-ΔRD, containing CckA codons 70–295 (Supplementary Table 1). To construct pJAB41, the pET28b(+) expression plasmid was digested with NdeI and SacI restriction enzymes. An insert containing CckA codons 70–295 was then PCR amplified using primers JABp64 and JABp100, which contained NdeI and SacI restriction sites in their 5′ overhangs, respectively. The insert was then digested with NdeI and SacI, and ligated into the digested pET28b(+). The following plasmids were constructed in a similar manner: pJAB27, pJAB41–47.

The plasmid pTM19 was generated via Gibson assembly and permits overexpression of CckA ΔPAS-B, containing CckA codons 70–182 fused directly to codons 296–691 (Supplementary Table 1). To construct pTM19, the pTEV5 expression plasmid was first digested with NheI (Supplementary Table 2). Two inserts were generated via PCR: CckA 70–182 was amplified using primers tmp56 and tmp57, and CckA 296–691 was amplified using primers tmp58 and tmp59 (Supplementary Table 3). Each insert contained 5′ and 3′ overhangs that allowed for their complementary pairing to each other and with the pTEV5 backbone. The following plasmids were constructed in a similar manner: pTM19, 28, 31–33, 38 and pAP433.

The construction of plasmids pTEV5 (ref. 63), pJAB27 (ref. 17), pWSC30 (ref. 9) and pRVYFPC-6 (ref. 64) were described previously. The strains LS101 and WSC229 (ref. 9), and JAB70 were described previously.

The CckA-eYFP mutant strains were constructed by electroporating plasmids pTM28 and 31–33 into NA1000 cells, and then cells were selected for chloramphenicol resistance. The native, chromosomal copy of CckA was then deleted by transduction, using ΦCr30 phage to move a gentamycin resistance-marked deletion of *cckA* (from strain LS3382) into the merodiploid strains. Gentamycin/chloramphenicol-resistant colonies were then re-streaked onto gentamycin/chloramphenicol PYE plates. Colonies that grew on the second round of plates were screened by PCR for replacement of the native copy of CckA with the gentamycin resistance cassette. We were unable to delete native *cckA* in a strain carrying the ΔPAS-A ΔPAS-B CckA-eYFP plasmid in four transduction attempts, implying that deletion of both PAS domains simultaneously is lethal. The strains (THM115 and THM125) carrying ΔPAS-B CckA-eYFP as its only copy of CckA grew in M2G but not in PYE liquid culture.

In these CckA-eYFP mutant replacement strains, we also replaced the chromosomal copy of stpX with stpX::stpX-mCherry. This replacement was performed by transducing the kanamycin resistance-marked stpX-mCherry replacement from the strain AP501. Transductants were selected via kanamycin resistance on M2G plates. The complete tables of strains, plasmids and oligonucleotides can be found in the Supplementary Information.

### Protein purification

CckA 70–691 (WT), 70–544 (ΔRD), 197–691 (ΔPAS-A), 296–691 (ΔPAS-A-ΔPAS-B), 70–182; 296–691 (ΔPAS-B), 70–378 (ΔCA-ΔRD), 70–295 (ΔDHp-ΔCA-ΔRD), 70–182; 296–544(ΔPAS-B-ΔRD), 197–544 (ΔPAS-A-ΔRD) and 296–544 (ΔPAS-A-ΔPAS-B-ΔRD) were expressed and purified as described previously, summarized below in ref. 17.

*E. coli* harbouring the expression plasmid were grown to OD-600 of 0.4 and switched to their expression temperatures of 24 °C for 30 min. Protein expression was induced for 3 h using 500 μM isopropyl-β-D-thiogalactoside. Cells were harvested by centrifugation and the cell pellets were stored at −80 °C.

Cell pellets were resuspended in lysis buffer (500 mM KCl, 50 mM HEPES-KOH pH 7.9, 1 mM dithiothreitol and 25 mM imidazole. One tablet of protease inhibitors (Roche) were added for every 50 ml of lysis buffer along with 0.3 μl benzonase nuclease (Sigma) per 1 l starting culture. Resuspended cells were lysed using three passes through an EmulsiFlex (Avestin). Insoluble material was pelleted at 29,000*g* for 45 min at 4 °C. The supernatant was decanted, and allowed to incubate with Ni-NTA agarose resin (1 ml NTA slurry/1 l culture) for 1 h at 4 °C. The Ni-NTA resin was washed prior to protein application using Ni-NTA wash buffer (500 mM KCl, 50 mM HEPES-KOH pH 7.9, 25 mM imidazole). The column flow-through was collected and the column was washed with three-column volumes of Ni-NTA wash buffer three times. The protein was eluted in 12 ml NTA elution buffer (500 mM KCl, 50 mM HEPES-KOH pH 7.9, 250 mM imidazole, 10% glycerol).

Samples requiring additional purification were buffer exchanged (PD-10 columns, GE Healthcare) into low-salt QA buffer (50 mM Tris pH 8.0, 50 mM KCl, 10% glycerol) for anion-exchange chromatography. The samples were then applied to a 1-ml HiTrap Q HP ion exchange column. The protein was eluted into 1.5 ml fractions using a linear gradient from 100% QA buffer to 100% QB buffer (50 mM Tris pH 8.0, 1 M KCl, 10% glycerol) over 30 ml at 1 ml min^−1^. Fractions containing protein were pooled.

Eluates from the nickel and anion-exchange columns were concentrated using Amicon spin filters. The concentrated proteins were then dialyzed overnight into kinase storage buffer (200 mM KCl, 50 mM HEPES-KOH pH 8.0, 10% glycerol), aliquoted and stored at −80 °C. CckA 195–295 was expressed overnight at 20 °C with 500 μM isopropyl-β-D-thiogalactoside. Protein purification was otherwise identical to the other CckA constructs.

### Production of Large Unilamellar Liposomes

Polar phospholipids (Avanti Polar Lipids; Alabaster, AL) dissolved in chloroform were mixed in a 9:1 mass ratio of 9 parts 1,2-dioleoyl-*sn*-glycero-3-phospho-(1′-*rac*-glycerol; sodium salt; di-oleoyl-phosphatidyl glycerol: product 840475, containing two 18-carbon acyl chains with a *cis*-alkene between carbon atoms 9 and 10 in each chain) to 1 part 1,2-dioleoyl-*sn*-glycero-3-[(N-(5-amino-1-carboxypentyl)iminodiacetic acid)succinyl] (nickel salt; DGS-NTA(Ni): product 790404C, containing two 18-carbon acyl chains with a *cis*-alkene between carbon atoms 9 and 10 in each chain). The *cis*-alkene acyl chains allow for membrane fluidity at room temperature. Lipid batches were prepared in 10 mg batches in a 10-ml roundbottom flask. Large unilamellar liposomes (LUVs) were produced as described, summarized here in refs 65, 66, 67. Briefly, the chloroform solvent was removed by rotovap at 20 mbar for 20 min to leave a dry, thin film on the flask. The film was then rehydrated to 10 mg ml^−1^ in liposome buffer (50 mM KCl, 10 mM HEPES-KOH pH 8.0, 10% glycerol v/v). The buffer was vortexed with the film for 10 min until the lipid was fully dissolved. The aqueous lipid mixture was then subjected to 10 freeze/thaw cycles in liquid nitrogen and a 37 °C water bath. The freeze-thaw mixture was then extruded for 11 passes through 100 nm pores of a polycarbonate filter using the Avanti Mini-Extruder. The resulting LUVs were collected in a glass vial and stored at 4 °C for up to 1 month.

### Radiolabelling phosphorylation assays

Radiolabelled autophosphorylation assays were similar to previous protocols^9^. CckA constructs (5 μM) were incubated in low-salt kinase buffer (50 mM KCl, 10 mM HEPES-KOH pH 8.0, 10% glycerol) with 5 mM MgCl_2_ and 0.5 mM ATP in 25 μl reaction volumes. Radiolabelled ATP was supplemented at 0.167 μCi μl^−1^ to give a total of 4.2 μCi [γ−^32^P] ATP per reaction. The reaction mixture was loaded onto 12% acrylamide gels and subjected to electrophoresis. Reaction time points were removed and quenched with 2 × Laemmli sample buffer. The reaction extent was measured by exposing a phosphor screen to the gels for 3 h, and the screen was subsequently imaged on a Typhoon storage phosphorimager (Molecular Dynamics). Band intensities were quantified using ImageJ.

We also adapted a method for measuring CckA reaction extent that uses nitrocellulose blots rather than gels to separate the protein from the crude reaction mixture^68^. The nitrocellulose blot offers higher throughput and sensitivity, allowing us to decrease the radiolabelled ATP to 0.07 μCi μl^−1^, for a total of 1.8 μCi [γ−^32^P] ATP per reaction. CckA reactions and quenching were otherwise performed identically to the previous protocol. The quenched mixture was spotted in 4 μl aliquots directly onto the nitrocellulose blot, and the bulk ATP and sample buffer were removed by rinsing the blot 6 times with 40 ml of 12.5 mM sodium pyrophosphate buffer, pH 10. The blot was then imaged on a phosphor screen as before.

For LUV-based assays, CckA was allowed to incubate for 20 min with varying amounts of LUVs before addition of ATP stocks. The LUVs were rehydrated in low-salt kinase buffer to avoid salt-dependent effects from titrating the amount of lipid in the reaction. For c-di-GMP inhibition assays, the concentration of MgCl_2_ was increased to 15 mM due to the observed dependence on MgCl_2_ for inhibition (Supplementary Fig. 10). Varying amounts of c-di-GMP were pre-incubated with CckA and MgCl_2_ in kinase buffer before addition of ATP.

### Biochemical data analysis

In Fig. 3a, the normalized CckA kinase activities were fit as a function of the number of CckA per liposome. The fit was of the form:


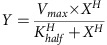


where *Y* is the normalized kinase activity, *X* is the number of CckA per liposome, *H* is the Hill slope and *K*_half_ is the number of CckA molecules per liposome that produces half-maximal velocity.

In Fig. 4b,c, the normalized CckA dose-response to c-di-GMP was fit as follows:


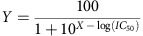


where *Y* is the normalized CckA kinase activity, *X* is the concentration of c-di-GMP in μM and the IC_50_ is the concentration of c-di-GMP that yielded 50% of its maximal response.

In Fig. 6a,b, the data were fit to a single exponential decay with a plateau at 20% maximum signal due to residual signal that did not decay over a 3-h period. The data shown are from the linear period of decay on the semi-log plot.

Data analysis was performed using Prism 6 (GraphPad).

### Surface plasmon resonance

The interaction of c-di-GMP with CckA variants was analysed by SPR using a BIACORE T200 biosensor system (GE Healthcare). C-di-GMP dilutions were performed in HEPES (50 mM HEPES at pH 8, 200 mM KCl, 5 mM MgCl_2_) running buffer. CckA variants were immobilized onto CM5 biosensor chip by amine coupling chemistry using *N*-hydroxysuccinimide and *N*′-(3-dimethylaminopropyl) carbodiimide hydrochloride. To investigate binding of c-di-GMP to CckA variants, the dilutions of c-di-GMP were injected over the different CckA variants surfaces. The experiments were performed at 25 °C using a flow rate of 50 μl min^−1^. For each experiment, at least 6 different concentrations of c-di-GMP were injected over each experimental and control flow cell for 60 s. Dissociation was allowed to occur at the same flow rate for 600 s in running buffer alone. All data were corrected for non-specific binding by subtracting the signal measured in a control cell lacking immobilized ligand. Both data processing and kinetic fitting were performed using BIAevaluation software v2.0 (GE Healthcare).

### RT-qPCR gene expression assays

The effects of the CckA mutations ΔPAS-A and ΔPAS-B on the transcription of CtrA-controlled genes were assayed by RT-qPCR. To obtain RNA samples, 0.5 ml of cells at an OD-600 of 0.3 were pelleted for 30 s at 15,000 r.p.m. in a benchtop centrifuge at room temperature. Cell pellets were then immediately flash frozen in liquid nitrogen. The pellets were then resuspended in TRIzol (Ambion) and extracted using Phase Lock Gel-Heavy tubes (5 PRIME), and RNA was purified using the RNA Clean and Concentrator-5 kit (Zymo Research). Genomic DNA was eliminated by treating the samples twice with 1 MBU Baseline-ZERO DNAse (Epicentre) at 37 °C for ∼20 min. RNA was recollected using Zymo-Spin columns and subsequently reverse transcribed using the SuperScript III reverse transcriptase kit (Invitrogen). Following reverse transcription, remaining RNA was degraded via RNAse H treatment, and the cDNA was diluted tenfold before beginning qPCR.

Expression was measured on an Applied Biosystems 7500 Fast Real-Time PCR system, using 7500 Software v 2.0.1. The 15-μl qPCR reaction contained 2 μl of cDNA, 7.5 μl of SYBR Green Dye master mix and 5.5 μl of primer mix. The primer mix contained the forward and reverse primers to form ∼100 bp amplicons in the genes of interest, at a final primer concentration in the reaction of 230 nM. Expression measurements were then made by comparing cycle-threshold (C_T_) of the amplicons of interest to an internal standard amplicon in the gene Rho, a house-keeping gene that is insensitive to CtrA cellular concentrations. As a negative control, we verified the removal of genomic DNA template contamination by measuring C_T_ of RNA samples not treated with reverse transcriptase. We additionally measured amplification of a genomic DNA standard curve to verify that the Rho, ccrM, sciP and divK amplicons formed unique products and with amplification efficiencies within 10% of one another at 92.3, 96.7, 87.0 and 89.4 per cent, respectively. Data was analysed using the delta–delta-C_T_ method^69^. Final gene expression measurements represent the average and s.d. of three biological replicates, each composed of at least two technical replicates.

### Microscopy

Cell cultures were grown overnight in M2G to an OD_600_ of 0.2–0.4, then immobilized onto a 1.5% agarose/M2G pad. Samples were imaged on a Leica DM 6000 B microscope with a HCX 100X PH3 1.40 NA 0.1 objective, Hamamatsu EM-CCD C9100 camera, and acquired through Metamorph software (Molecular Devices, version 7.7). Phase-contrast, YFP fluorescence and mCherry fluorescence images were recorded. Image threshold and gamma level were adjusted in Adobe Photoshop to clearly show both the polar accumulations and diffuse populations of CckA in the displayed micrographs. Quantitative analysis of fluorescence focus formation in images was performed using ImageJ's^70^ cell-counter (version 1.45s).

## Additional information

**How to cite this article:** Mann, T. H. *et al*. A cell cycle kinase with tandem sensory PAS domains integrates cell fate cues. *Nat. Commun.* 7:11454 doi: 10.1038/ncomms11454 (2016).

## Supplementary Material

Supplementary Information Supplementary Figures 1-11, Supplementary Tables 1-3, Supplementary Note 1 and Supplementary References
