# Supplementary material for: A cell cycle kinase with tandem sensory PAS domains integrates cell fate cues
Source: Nat Commun. 2016 Apr 27;7:11454. doi: 10.1038/ncomms11454 (PMC4853435; doi:10.1038/ncomms11454)
Supplement: Supplementary Information — Supplementary Figures 1-11, Supplementary Tables 1-3, Supplementary Note 1 and Supplementary References [file ncomms11454-s1.pdf]

Supplementary Figures

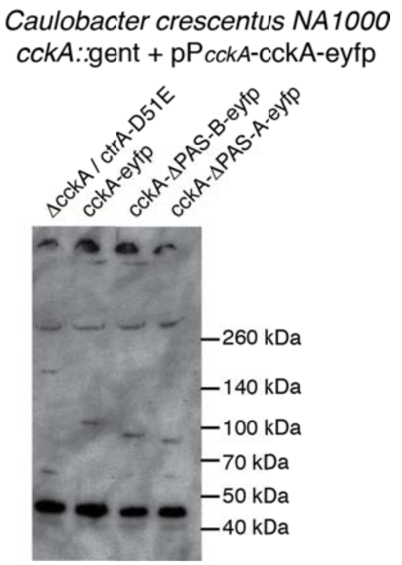

Supplementary Figure 1. CckA-eYFP constructs are stably expressed *in vivo*. Cells expressing plasmid-borne WT CckA-eYFP, CckA-eYFP ΔPAS-B, and CckA-eYFP ΔPAS-A as the sole copy of CckA were harvested at mid-log phase and normalized for cell-density. A strain with no copy of CckA, kept viable by the phosphomimetic mutation CtrA D51E, was used as a negative control. Cell lysates were denatured, subjected to SDS-PAGE, transferred to a PVDF membrane, and subsequently blotted for CckA-eYFP using an anti-CckA antibody. Bands were detected via chemiluminescence with a goat-anti-rabbit secondary antibody conjugated to horseradish peroxidase.

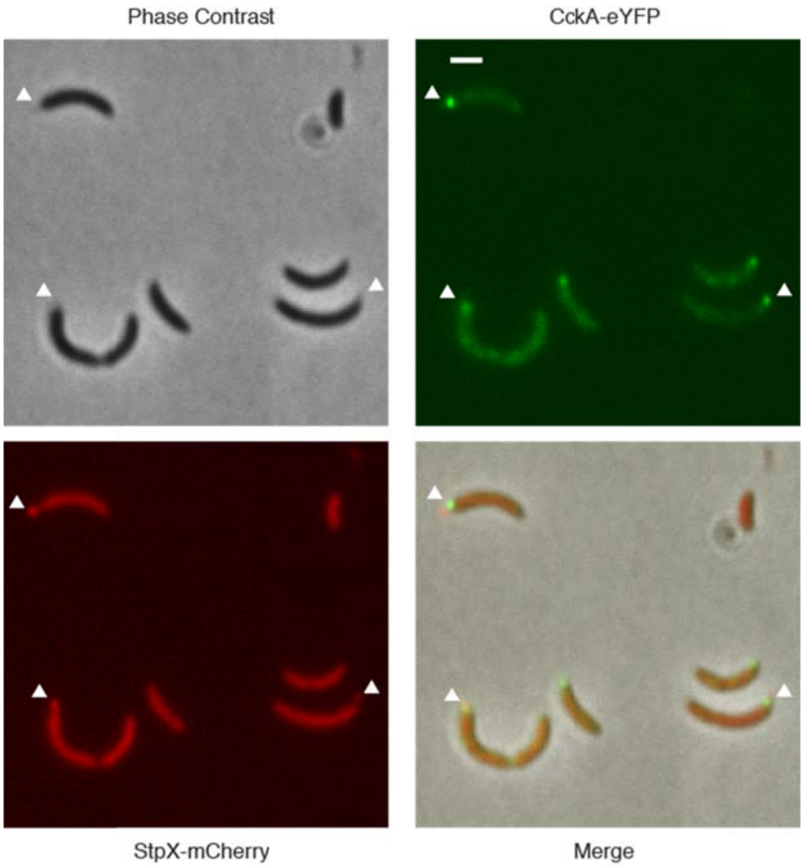

Supplementary Figure 2. StpX-mCherry functions as a polar marker for definitive identification of monopolar CckA-eYFP accumulations. A subcellular localization assay shows that plasmid-borne CckA can accumulate only at the stalked pole of the cell. The stalk pole protein StpX, which localizes to the stalk in addition to the cell body, can better illustrate the location of short stalks than phase contrast alone. CckA-eYFP was expressed from its native promoter on a low-copy replicating plasmid for each construct, with the chromosomal copy of CckA deleted. The heterogeneity of the low copy replicating plasmid segregation leads to a small fraction of cells not receiving the CckA-eYFP plasmid, as in the top right cell. StpX-mCherry was expressed as the sole copy from its native promoter on the chromosome. White arrowheads indicate the stalked pole in each image. Scale bar shown in eYFP panel, 2 µm, same for every panel.

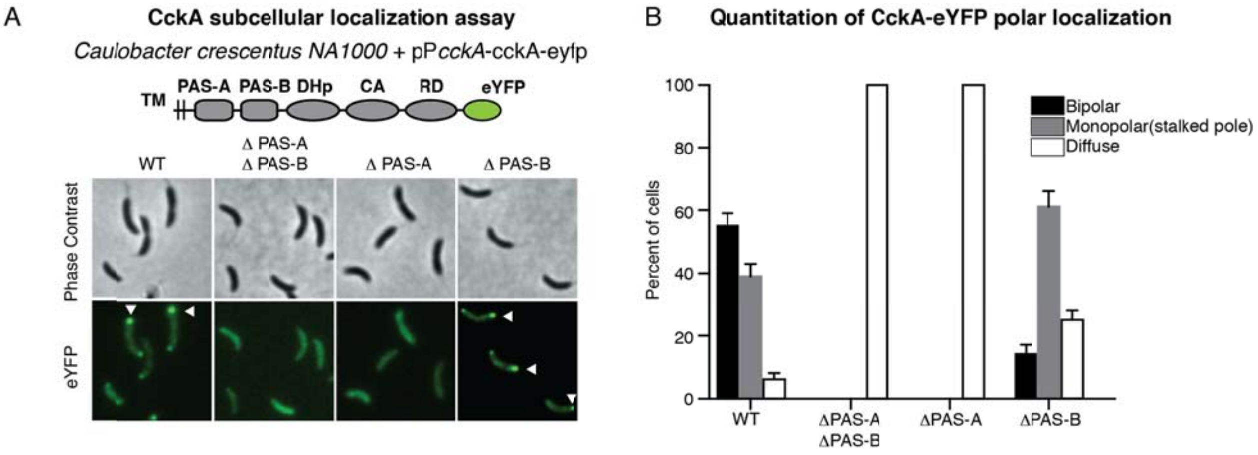

Supplementary Figure 3. CckA-eYFP requires its PAS domains for proper subcellular localization. (A) A subcellular localization assay shows that CckA's PAS domain A is necessary for polar accumulation *in vivo*. A cartoon representation the domain architecture of full-length CckA is shown with its N-terminal transmembrane tether and a C-terminal eYFP label. CckA-eYFP was expressed from its native promoter on a low-copy replicating plasmid for each construct. White arrowheads indicate the stalked pole in the eYFP image. (B) Quantitation of the proportion of cells in which CckA-eYFP localized to both cell poles (black bar), formed a single focus at the stalked pole (gray bar), or remained diffuse over the membrane (white bar). Deletion of PAS-B resulted in a shift towards stalked pole localization, while deletion of PAS-A totally abrogated subcellular accumulation at the poles. Error bars represent the standard deviation of three representative fields of cells (N > 400 cells for each construct).

```
                  70        80        90        100       110       120
Residue           |         |         |         |         |         |
CckA             RGSALSGGDADQAEGFIEALAEPAALAAADGRVLAANGPWREVMGEQRRLPKGVAGS
SS PSIPRED          HH    HHHHHHHHH     EEEE    EEEH  HHHHHHH   HHHH     H
Confidence       941000256899999996174013999899847233599999868786775587720

                     130       140       150       160       170       180
Residue              |         |         |         |         |         |
CckA             SLFAALVQARQGQMAEGMLSAGGTDYTAKVSRLAGGRLMIRLAPIVVAEPVVEDAS
SS PSIPRED       H      HHHHHHHHHHHHH     EEEEEEE    EEEEEEEEEEE   EEEEHH
Confidence       02453213455536888874378521578884289559999921110121156406
```

Supplementary Figure 4. The central beta-sheet of PAS-A may contain a critical polar localization element. The predicted secondary structure for PAS-A is shown. Vertical bars mark every ten residues, with codon numbering based on the full-length protein. Below the CckA codon sequence, the secondary structure PSIPRED[1] alpha-helix (H) or beta-sheet (E) prediction for each residue is marked. The confidence is scored from 0 to 9. The structure prediction shows that residues 152-180 are predicted to participate in the central beta-sheet that defines PAS domains. Previous observations showed that deletion of residues 80-149 led to partial, but incomplete, disruption of polar localization[2], indicating that this beta-sheet may contain a polar localization element.

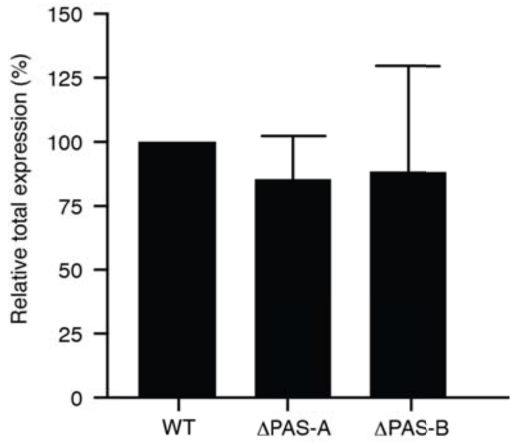

Supplementary Figure 5. Expression of Rho is consistent across CckA-eYFP variants. RT-qPCR assays of the expression of the transcription terminating gene Rho show that total expression of Rho remains consistent between CckA-eYFP variant strains, consistent with its promoter lacking a CtrA-binding motif. Expression was quantified by comparing the threshold cycles (C_T) for Rho between strains. Error bars represent the standard deviation of three biological replicates, each composed of at least two technical replicates.

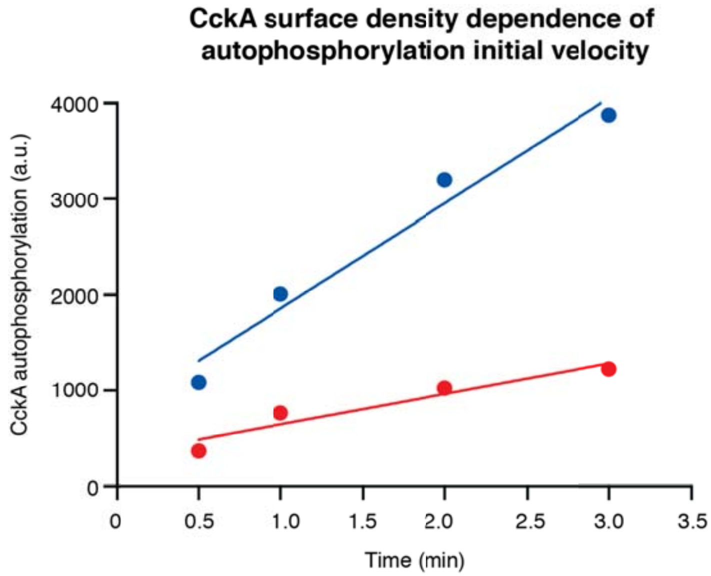

Supplementary Figure 6. CckA autophosphorylation initial velocity increases with increasing CckA surface density. Purified 5 μM CckA WT was incubated with 0.5 mM ATP and 4.2 μCi [γ–^{32}P] ATP at different CckA surface densities on liposomes. Autophosphorylation was assayed during the initial, linear phase of the reaction (0.5, 1, 2, 3 minutes). Samples were quenched into SDS sample buffer, blotted onto nitrocellulose and followed by phosphorimaging. Example data is shown for CckA at 800 (blue) and 200 (red) CckA molecules per liposome.

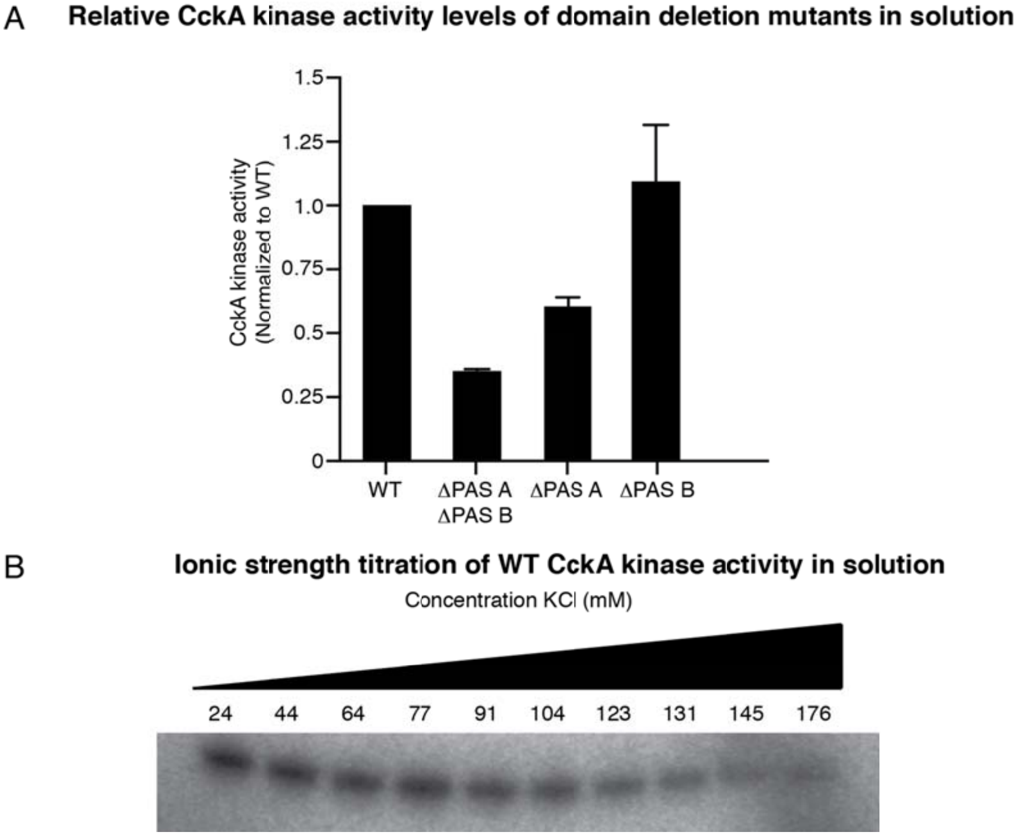

Supplementary Figure 7. CckA PAS domain deletions are active in solution, and CckA kinase activity is highly sensitive to ionic strength. (A) A panel of purified CckA domain deletion mutants was assayed for kinase activity in solution for a reaction period of three minutes. All four constructs were functional and capable of autophosphorylation. Error bars correspond to the range of two experiments. (B) WT CckA kinase activity is higher in low salt

solutions than in high salt solutions. Purified WT CckA at 3 μM was incubated with different concentrations of KCl in solution for 10 minutes, and autophosphorylation was initiated by the addition of [γ–^{32}P] ATP. After a reaction period of 8 minutes, samples were quenched into SDS sample buffer and subjected to gel electrophoresis. Autophosphorylation was measured by phosphorimaging.

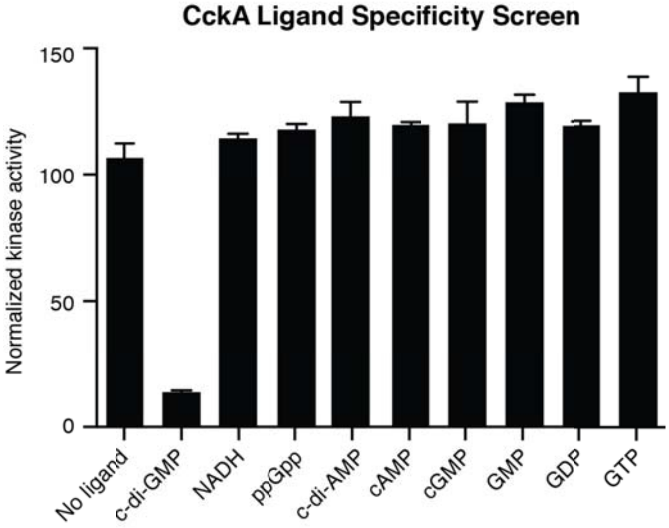

Supplementary Figure 8. Inhibition of CckA is specific for c-di-GMP. Purified CckA 5 μM was incubated in the presence of 0.5 mM ATP and 4.2 μCi [γ–^{32}P] ATP for 15 minutes in the presence of potential cytosolic small molecules (NADH, ppGpp) and chemically related species to c-di-GMP (c-di-AMP and others), each at 250 μM. Samples were quenched into SDS sample buffer, blotted onto nitrocellulose and followed by phosphorimaging. Error bars represent the standard deviation of three experiments.

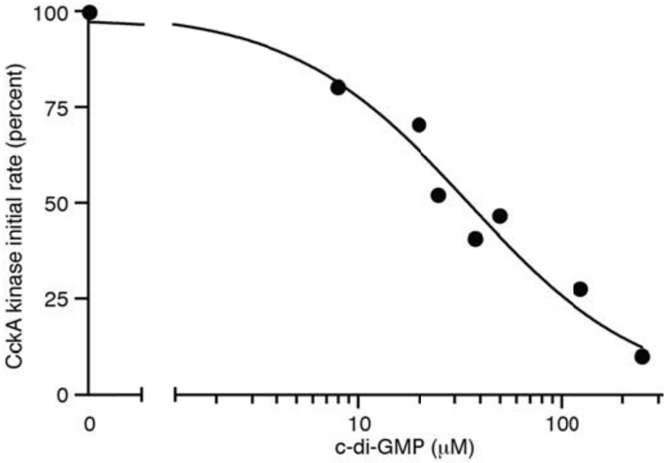

Supplementary Figure 9. C-di-GMP inhibits CckA autophosphorylation initial rate. C-di-GMP inhibits CckA kinase activity in an initial rate assay. Purified 5 µM CckA WT was incubated with [γ–^{32}P] ATP and increasing concentrations of c-di-GMP, and autophosphorylation was assayed during the initial, linear phase of the reaction (0.5, 1, 1.5, 2, 3 minutes). Samples were quenched into SDS sample buffer, blotted onto nitrocellulose and followed by phosphorimaging. Activity is normalized to the zero c-di-GMP condition. The data provided are from one experiment.

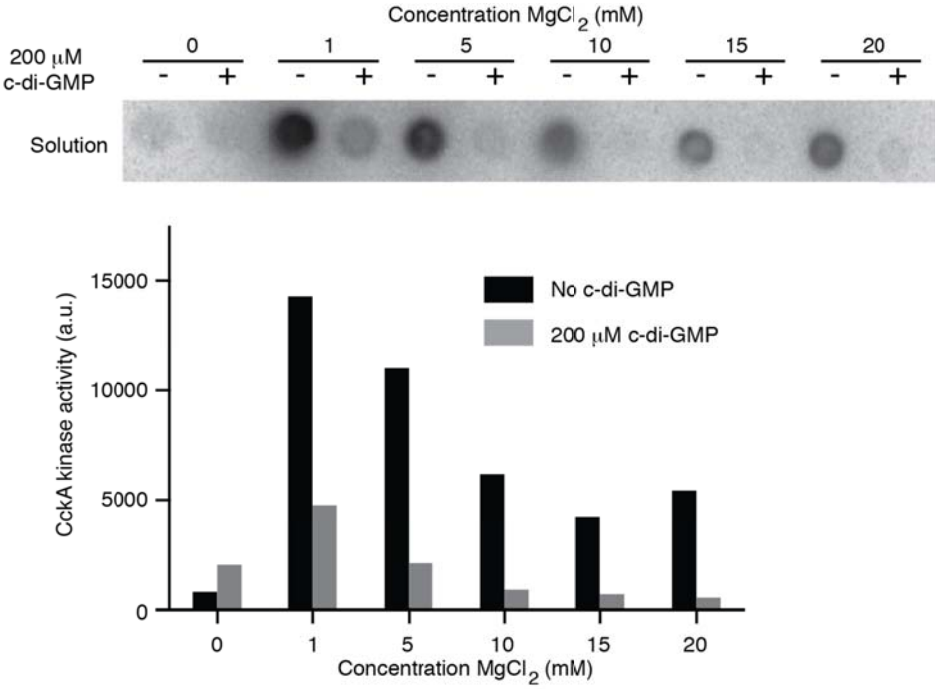

Supplementary Figure 10. C-di-GMP inhibition of CckA is magnesium-dependent. CckA in kinase buffer was allowed to pre-incubate with $MgCl_2$ and with (gray) or without (black bars) 200 μM c-di-GMP for 15 minutes. The final concentration of CckA in solution was 5 μM. CckA was then incubated with [γ–^{32}P] ATP for 8 minutes. Samples were quenched into SDS sample buffer, blotted onto nitrocellulose and followed by phosphorimaging. Raw data from the nitrocellulose membrane capture assay is shown. While Mg^{2+} was necessary for CckA kinase activity, increasing Mg^{2+} past 1 mM resulted in decreased kinase activity but also increased potency of c-di-GMP inhibition. The data provided are from a single experiment.

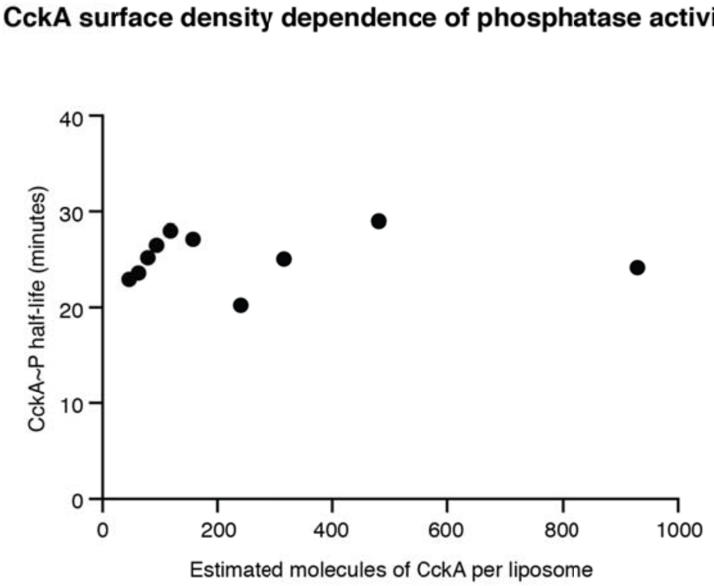

Supplementary Figure 11. CckA surface density effects on CckA~P half-life are minimal. CckA~P half-life was assayed by allowing the protein to phosphorylate in solution prior to purification away from ATP. CckA~P was then titrated at 5 mM onto increasing amounts of liposomes, creating a range of CckA~P surface densities. Dephosphorylation was monitored by quenching samples in SDS sample buffer at time points from 0 to 70 minutes after addition to the liposomes. Extent of phosphorylation was assayed by blotting the quenched reactions onto nitrocellulose and the blot was measured by phosphorimaging. CckA half-lives were measured by fitting the data to a single exponential decay. CckA~P half-life was approximately unchanged over a 19-fold CckA surface density range from approximately 50 to 950 CckA molecules per liposome, with a potentially mild linear increase from 23 to 28 minutes at low density. The data provided are from a single experiment.

Supplementary table 1 Plasmids and strains used in this study.

Plasmid	Description	Reference
pET-28b(+)	bacterial expression vector	Novagen
pTEV5	bacterial expression vector	[3]
pJAB27	pET-28b(+) CckA(70-691) expression vector	[4]
pJAB41	pET-28b(+) CckA(70-295) expression vector	This Study
pJAB42	pET-28b(+) CckA(70-378) expression vector	This Study
pJAB43	pET-28b(+) CckA(70-544) expression vector	This Study
pJAB44	pET-28b(+) CckA(197-544) expression vector	This Study
pJAB45	pET-28b(+) CckA(294-544) expression vector	This Study
pJAB46	pET-28b(+) CckA(197-691) expression vector	This Study
pJAB47	pET-28b(+) CckA(294-691) expression vector	This Study
pTM19	pTEV5-CckA(70-182; fused to 296-691) expression vector	This Study
pTM38	pTEV5-CckA(70-182;296-544) expression vector	This Study
pWSC30	pET-28b(+) PleC(310-842) expression vector	[5]
pAP433	pTEV5-DivJ(188-595) expression vector	Perez et al., in preparation
pAP500	pCHYC-2 stpX	Perez et al., in preparation
pRVYFPC-6	Low copy, replicating, C-terminal eyfp fusion vector	[6]
pTM28	pRVYFPC-6 CckA(1-691)	This Study
pTM31	pRVYFPC-6 CckA(1-69; fused to 197-691)	This Study
pTM32	pRVYFPC-6 CckA(1-69; fused to 294-691)	This Study
pTM33	pRVYFPC-6 CckA(1-182; fused to 296-691)	This Study

Strain	Description	Reference
C. crescentus NA1000	Laboratory Caulobacter crescentus strain	[5]
E. coli DH5α	Bacterial cloning strain	Invitrogen
E. coli Rosetta(DE3)pLysS	Bacterial expression strain	Novagen
E. coli BL21	Bacterial expression strain	Novagen
LS3382	NA1000 cckA::gent + pMR10-cckA	[7]
WSC229	pET-28b(+) PleC expression vector	[5]
AP434	BL21 pTEV5 DivJ(188-595)	Perez et al., in preparation
AP501	NA1000 stpX::stpX-mCherry	Perez et al., in preparation
JAB70	Rosetta pET-28b(+) CckA(70-691) expression vector	[4]
JAB97	Rosetta pET-28b(+) CckA(70-295) expression vector	This Study
JAB98	Rosetta pET-28b(+) CckA(70-378) expression vector	This Study
JAB99	Rosetta pET-28b(+) CckA(70-544) expression vector	This Study
JAB100	Rosetta pET-28b(+) CckA(197-544) expression vector	This Study
JAB101	Rosetta pET-28b(+) CckA(294-544) expression vector	This Study
WSC282	Rosetta pET-28b(+) CckA(197-691) expression vector	This Study
WSC283	Rosetta pET-28b(+) CckA(294-691) expression vector	This Study
THM48	BL21 pTEV5-CckA(70-182; fused to 296-691) expression vector	This Study
THM68	BL21 pTEV5-CckA(70-182; fused to 296-544) expression vector	This Study
AP434	BL21 pTEV5-DivJ(188-595) expression vector	Perez et al., in preparation
THM60	NA1000 + pRVYFPC-6 CckA(1-691)	This Study
THM72	NA1000 + pRVYFPC-6 CckA(1-69; fused to 197-691)	This Study

THM74	NA1000 + pRVYFPC-6 CckA(1-69; fused to 294-691)	This Study
THM70	NA1000 + pRVYFPC-6 CckA(1-182; fused to 296-691)	This Study
THM114	THM60 + cckA::gent transduction	This Study
THM115	THM70 + cckA::gent transduction	This Study
THM116	THM72 + cckA::gent transduction	This Study
THM125	THM115 + stpX::stpX-mCherry transduction	This Study
THM126	THM116 + stpX::stpX-mCherry transduction	This Study
THM127	THM114 + stpX::stpX-mCherry transduction	This Study

Supplementary table 2. Gibson cloning strategy to generate constructs.

Plasmid#	Strain#	Assembly method	Vector backbone	Restriction enzyme(s)	Vector insert(s)	Forward primer	Reverse primer	Note
pTM19	THM48	Gibson	pTEV5	NheI	CckA(70-182)	tmp56	tmp57	
					CckA(296-691)	tmp58	tmp59	
pTM38	THM68	Gibson	pTEV5	NheI	CckA(70-182; fused to 296-544)	tmp56	tmp119	*
pTM28	THM60	Gibson	pRVYFPC-6	SacI, HindIII	CckA promoter	tmp81	tmp75	**
					CckA(1-691)	tmp74	tmp75	
pTM31	THM72	Gibson	pRVYFPC-6	SacI, HindIII	CckA promoter + (1-69)	tmp81	tmp90	**
					CckA (197-691)	tmp91	tmp80	
pTM32	THM74	Gibson	pRVYFPC-6	SacI, HindIII	CckA promoter + (1-69)	tmp81	tmp92	**
					CckA (294-691)	tmp93	tmp75	
pTM33	THM70	Gibson	pRVYFPC-6	SacI, HindIII	CckA Promoter + (1-182)	tmp81	tmp94	**
					CckA (296-691)	tmp58	tmp75	

* The PCR template was pTM19.

** The vanillate region was removed via double digest and CckA was expressed under its native promoter.

Supplementary table 3. DNA oligonucleotides used in this study.

Oligo	Plasmid(s)	Description	Site	Sequence 5'-3' (Restriction site is underlined.)
JABp64	pJAB27<br>pJAB41<br>pJAB42<br>pJAB43	CckA70-691 forward	NdeI	GGCCTTGTCCATATGCGCGGCTCAGCGCTTTCCGG
JABp66	pJAB27<br>pJAB46<br>pJAB47	CckA70-691 reverse	SacI	ACTGCGGAGCTCCTACGCCGCCTGCAGCTGCTG
JABp99	pJAB44<br>pJAB46	CckA 197 forward	NdeI	ACCCCATATGCTGGACGCCTTCGC
JABp100	pJAB41	CckA 295 reverse	SacI	ATCTGGAGCTCCTaGGACACGTCGATCATG
JABp101	pJAB45<br>pJAB47	CckA 294 forward	NdeI	ACATGCATATGGTGTCCGAGCAGAAGCAGATCG
JABp102	pJAB42	cckA 70-378 reverse	SacI	ACGAGCTCCTaCACGGTCTGCTTGCGCGA
JABp104	pJAB43<br>pJAB44<br>pJAB45	CckA70-544 reverse	SacI	CCGAGCTCCtATTCATAGACCGGCAGG
tmp56	pTM19<br>pTM38	CckA 70 forward		CCAACTAGTGAAAACCTGTATTTTCAGGGCGCTCGCGGCTCAGCGCTTTCC
tmp57	pTM19	CckA 182 reverse		TCTGCTTCTGCTCCGACGCGTCTTCCACAACC
tmp58	pTM19<br>pTM33	CckA 296 forward		GGAAGACGCGTCGGAGCAGAAGCAGATCGAGCTG
tmp59	pTM19	CckA 691 reverse		GCTCGAGAATTCCATGGCCATATGGCTTTACGCCGCCTGCAGCTG
tmp73	pTM28	PcckA reverse		GCAAGTCGGCCATCGGCGAGGTTGTACCTTTCTTACGGC
tmp74	pTM28	CckA 1 forward		TACAACCTCGCCGATGGCCGACTTGCAGCTCCAGG
tmp75	pTM28<br>pTM32<br>pTM33	CckA 691 reverse		CGCGTAACGTTCGAATTCTCCGGAGCCGCCGCCTGCAGCTGCTGC
tmp80	pTM31	CckA 691 reverse		CGCGTAACGTTCGAATTCTCCGGAGCCGCCGCCTGCAGCTGCTG
tmp81	pTM28<br>pTM31<br>pTM32<br>pTM33	PcckA forward		GCTTAATGAATTACAACAGTTTTTATATAAGCTTTCATTCTCCAGAAAACTGACCTAGCCTCC
tmp90	pTM31	CckA 69 reverse		CGAAGGCGTCCAGAATGGCGACAAGGCCCAACACA
tmp91	pTM31	CckA 197 forward		CCTTGTCGCCATTCTGGACGCCTTCGCCGGAGC
tmp92	pTM32	CckA 69 reverse		GCTTCTGCTCGGACACAATGGCGACAAGGCCCAACACA
tmp93	pTM32	CckA 294 forward		CCTTGTCGCCATTGTGTCCGAGCAGAAGCAGATCGAGC
tmp94	pTM33	CckA 182 rev		CGATCTGCTTCTGCTCCGACGCGTCTTCCACAACCGG
tmp119	pTM38	CckA 544 rev		GCTCGAGAATTCCATGGCCATATGGCTCTATTCATAGACCGGCAGGAAGATGC

oap125	pAP433	DivJ 188 forward		AAACCTGTATTTTCAGGGCGCTAGCGCCAGCG AGATCATCGGTCTG
oap127	pAP433	DivJ 595 reverse		GAGAATTCCATGGCCATATGGCTAGCCTAGC GCGGCGCAAAGGC
WSCp114	pWSC30	PleC 310 forward	NheI	AAAAGCTAGCGTCGCCCATCGCGAGTTCATC G
WSCp115	pWSC30	PleC 842 reverse	SacI	AAAAGAGCTCCCTCAGGCCGCCACGAAGTC
tmp189		qpcr_ccrM1		GCCGACCGTGATCGAGCCG
tmp190		qpcr_ccrM2		GGCACCATCGTCGAGGC
tmp197		qpcr_divK1		GCAGGCGCTTGATGGTC
tmp198		qpcr_divK2		GGAGCGCATCCGCGAG
tmp201		qpcr_sciP1		GGTCTGCTCTCGCTCGA
tmp202		qpcr_sciP2		GCCAGGCCGTGCCGA
mdm306		qpcr_rho1*[8]		GTCGAGAACGCCAACTCCAT
mdm307		qpcr_rho2		CGAGGGTCTTCAGGATCGC

Supplementary note 1. Calculation of the number of CckA molecules per liposome and the density of CckA per unit area.

To calculate the number of binding sites for CckA on a liposome, we can start by computing the total surface area of the liposome. Extrusion of DOPG liposomes through 100 nm pores yields liposomes with vesicle diameters of 97-106 nm[9], approximated here as 100 nm.

100 nm diameter sphere – radius = 500 Å

Surface area (SA) of a sphere = $4\pi r^2$

SA = $3.14 * 10^6$ Å^2

The surface area occupied by each lipid headgroup varies by headgroup identity, acyl chain length, and temperature[10]. For 18:1 cis-DOPG, comprising 90% of the lipid mass of the liposomes presented in this study, each headgroup occupies 69.4 Å^2 at 20°C and 70.8 Å^2 at 30°C[11].

To calculate the lipids on the outer layer, we will assume similar area on average per (69.5 Å^2) lipid despite 10% NTA lipid incorporation.

lipids outer membrane = SA / (area per lipid headgroup)

lipids = $3.14 * 10^6$ Å^2 / (69.5 Å^2/lipid) = $4.52 * 10^4$ lipids.

There are approximately 45,000 on the outer layer. Of these, 10% (4,500) are DGS-NTA sites for possible CckA attachment.

CckA loading onto liposomes could be limited by either the number of NTA sites or by the total surface area of the liposome.

Calctool.org estimates a 622-residue protein (WT CckA) at 5.9 nm diameter. This calculation assumes that the protein is spherical.

Projecting the sphere down onto the surface of the liposome, we can calculate the surface area of one molecule of CckA as a circle of radius ~3 nm.

Area of circle (A) = πr^2

A = 2,800 Å^2 – this is the area on the liposome surface that the protein occupies.

SA liposome / $A_{protein}$ = number of proteins that can fit.

3.14 * 10^6 Å^2 / 2,800 Å^2 = 1100 proteins

Given that 1,100 proteins can fit by the surface area calculation and there are 4,500 NTA sites available, protein loading on the liposome is surface-area limited.

To determine the molar ratio between CckA molecules and liposome particles, we need to compute the mass of a single liposome. The mass of the lipids in the liposome will be equal to the sum of the inner and outer sheets of the bilayer.

Bilayer thickness = 3.6 nm

Inner diameter of liposome = ~96 nm.

Lipids on inner layer:

Inner SA / 69.5 Å^2 = 4.2 * 10^4 lipids on inner layer

Total lipid molecules (TL) = #lipids inner + #lipids outer layer

TL = (4.2 + 4.5) * 10^4 = 8.7 * 10^4 lipids/liposome

We used 10% DGS-NTA lipids and 90% DOPG lipids by mass. The molecular weights of the lipids are similar, so here we use their mass ratios as a proxy for their molar ratios.

Mass of liposome = (mass NTA lipids) * (#NTA) + (mass DOPG) * (#DOPG)

8.7 * 10^3 NTA lipids, 1057 g/mol → 1.53 * 10^{-17} g/liposome

7.9*10^4 DOPG lipids, 797 g/mol → 1.05*10^{-16} g/liposome

Total mass of one liposome = 1.20 * 10^{-16} g/liposome

For a 5 μM CckA sample in a 25 μL volume, we use 8.5 μg protein.

If we add an equal mass of lipid to protein, we reach approximately maximum loading capacity.

#Liposomes = total mass liposomes / mass per liposome

#Liposomes in reaction = 8.5 * 10^{-6} g liposomes / (1.2 * 10^{-16}) g/liposome = 7.1*10^{10} liposomes

#CckA molecules in the reaction = 7.5 * 10^13

CckA molecules per liposome = 1100 (maximum number of CckA sites by surface area).

Supplementary References

1. Jones, D. T. Protein secondary structure prediction based on position-specific scoring matrices. *J. Mol. Biol.* **292,** 195–202 (1999).
2. Angelastro, P. S., Sliusarenko, O. & Jacobs-Wagner, C. Polar localization of the CckA histidine kinase and cell cycle periodicity of the essential master regulator CtrA in Caulobacter crescentus. *J. Bacteriol.* **192,** 539–52 (2010).
3. Rocco, C. J., Dennison, K. L., Klenchin, V. a., Rayment, I. & Escalante-Semerena, J. C. Construction and use of new cloning vectors for the rapid isolation of recombinant proteins from Escherichia coli. *Plasmid* **59,** 231–237 (2008).
4. Blair, J. a *et al.* Branched signal wiring of an essential bacterial cell-cycle phosphotransfer protein. *Structure* **21,** 1590–601 (2013).
5. Childers, W. S. *et al.* Cell fate regulation governed by a repurposed bacterial histidine kinase. *PLoS Biol.* **12,** e1001979 (2014).
6. Thanbichler, M., Iniesta, A. a & Shapiro, L. A comprehensive set of plasmids for vanillate- and xylose-inducible gene expression in Caulobacter crescentus. *Nucleic Acids Res.* **35,** e137 (2007).
7. Jacobs, C., Domian, I. J., Maddock, J. R. & Shapiro, L. Cell cycle-dependent polar localization of an essential bacterial histidine kinase that controls DNA replication and cell division. *Cell* **97,** 111–20 (1999).
8. Mazzon, R. R., Lang, E. a S., Silva, C. a P. T. & Marques, M. V. Cold shock genes cspA and cspB from Caulobacter crescentus are posttranscriptionally regulated and important for cold adaptation. *J. Bacteriol.* **194,** 6507–17 (2012).
9. Ertel, A., Marangoni, A. G., Marsh, J., Hallett, F. R. & Woodt, J. M. Mechanical properties of vesicles 1 . Coordinated analyses of osmotic swelling and lysis vesicles : DLS spectroscopy. *Biophys. J.* **64,** (1993).
10. Petrache, H. I., Dodd, S. W. & Brown, M. F. Phosphatidylcholines Determined by 2 H NMR Spectroscopy. *Biophys. J.* **79,** 3172–3192 (2000).

11. Pan, J. Molecular structures of fluid phase phosphatidylglycerol bilayers as determined by small angle neutron and X-ray scattering. *Biochim. Biophys. Acta* **1818,** 2135–2148 (2012).
